# Programming cell-free biosensors with DNA strand displacement circuits

**DOI:** 10.1038/s41589-021-00962-9

**Published:** 2022-02-17

**Authors:** Jaeyoung K. Jung, Chloé M. Archuleta, Khalid K. Alam, Julius B. Lucks

**Affiliations:** 1 Department of Chemical and Biological Engineering, Northwestern University, Evanston, IL USA; 2 Center for Synthetic Biology, Northwestern University, Evanston, IL USA; 3 Center for Water Research, Northwestern University, Evanston, IL USA; 4 Stemloop, Inc., Evanston, IL USA; 5 Interdisciplinary Biological Sciences Graduate Program, Northwestern University, Evanston, IL USA

**Keywords:** DNA, RNA, Transcription, Synthetic biology

## Abstract

Cell-free biosensors are powerful platforms for monitoring human and environmental health. Here, we expand their capabilities by interfacing them with toehold-mediated strand displacement circuits, a dynamic DNA nanotechnology that enables molecular computation through programmable interactions between nucleic acid strands. We develop design rules for interfacing a small molecule sensing platform called ROSALIND with toehold-mediated strand displacement to construct hybrid RNA–DNA circuits that allow fine-tuning of reaction kinetics. We use these design rules to build 12 different circuits that implement a range of logic functions (NOT, OR, AND, IMPLY, NOR, NIMPLY, NAND). Finally, we demonstrate a circuit that acts like an analog-to-digital converter to create a series of binary outputs that encode the concentration range of the molecule being detected. We believe this work establishes a pathway to create ‘smart’ diagnostics that use molecular computations to enhance the speed and utility of biosensors.

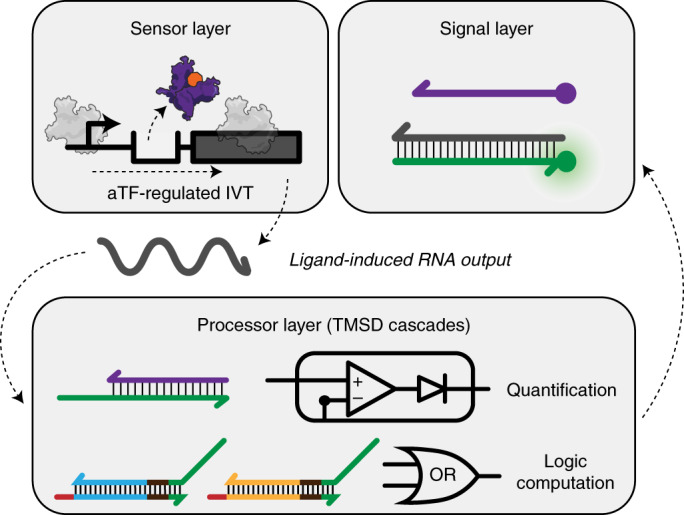

## Abstract (web summary)

Equipping ROSALIND, a cell-free biosensing platform, with information processing circuits based on toehold-mediated DNA strand displacement enhances sensor performance and enables logic gate computation.

## Main

**Fig. 1:**
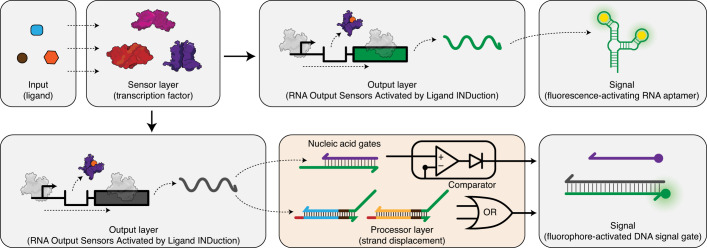
Interfacing cell-free biosensors with DNA strand displacement circuits expands and enhances their function. (Upper) A cell-free biosensor typically activates when a target compound (input) binds to a protein transcription factor (sensor layer) that is configured to activate expression of a reporter construct (output layer). This results in the production of a detectable signal such as fluorescence. (Lower) Adding a downstream information processing layer before signal generation can enhance the performance and expand the function of cell-free biosensors by adding computational features such as logic processing and signal comparison. Here, this is implemented by wiring the biosensing output layer to produce a single-stranded RNA capable of activating toehold-mediated strand displacement circuits that generate signal.

Cell-free biosensing is emerging as a low-cost, easy-to-use and field-deployable diagnostic technology platform that can detect a range of chemical compounds related to human and environmental health^1,2^. At their core, these systems consist of two layers: an RNA or protein-based biosensing layer and a reporter construct output layer. By genetically wiring these layers, a signal is generated when the target compound binds to the biosensor and activates reporter expression (Fig. 1). Reactions are assembled by embedding these layers within cell-free systems and freeze-drying them for easy storage, transportation and rehydration with a sample of interest at the point-of-need^1,3^. Using this approach, cell-free biosensors have successfully detected compounds related to human health such as zinc^4^ and quorum sensing molecules from pathogenic bacteria^5^, drugs such as gamma-hydroxybutyrate^6^ and water contaminants such as fluoride^1^, atrazine^2^, antibiotics and heavy metals^7^.

However, existing cell-free biosensors often lack an information processing layer that can manipulate responses from the sensing layer before signal generation (Fig. 1). Such information processing layers are a natural feature of organisms and enable cells to activate stress responses, guide development and make behavioral decisions on the basis of intracellular and extracellular cues^8^. For this reason, genetic information processing layers that implement logic and feedback have been extensively leveraged and engineered in synthetic cellular systems^9,10^. Similarly, we have previously shown that RNA-based circuits can be added to a cell-free biosensors platform called RNA Output Sensors Activated by Ligand INDuction (ROSALIND) to improve their specificity and sensitivity without engineering the protein biosensors^7^. However, these circuits still directly act on either the sensing or the output layer, limiting our ability to improve and expand their function.

Here, we develop a generalizable information processing layer to enhance and expand the function of ROSALIND by leveraging toehold-mediated DNA strand displacement (TMSD)—a computationally powerful DNA nanotechnology that can process molecular information in vitro^11^. In TMSD, single-stranded DNA (ssDNA) inputs exchange strands with double-stranded DNA ‘gates’ via complementary base pairing interactions to produce ssDNA output strands. By configuring DNA gates into different network architectures, a range of operations can be performed such as signal restoration^12^, signal amplification^13^ and logic computation^14,15^, much like a general chemical computational architecture^16^. The well-characterized thermodynamics of DNA base pairing enable large networks to be built from simple building blocks. In addition, reaction kinetics can be precisely tuned by changing the strength of the ‘toeholds’—single-stranded regions within the DNA gates that initiate the strand displacement process^17^. TMSD has led to the development of powerful devices including in vitro oscillators^18^, catalytic amplifiers^19^, autonomous molecular motors^20,21^ and reprogrammable DNA nanostructures^22,23^. Thus, there is a great potential for TMSD-based information processing to improve cell-free biosensors.

Although TMSD circuits have been used to detect nucleic acid targets such as microRNAs^24,25^ and human pathogens^26^, there are currently no general design rules for triggering TMSD circuits with small molecules to enable their use in cell-free biosensors. We therefore sought to create an interface that can convert the binding event of a chemical target to changes in nucleic acid strands that can trigger TMSD cascades. Allosteric transcription factors (aTFs) naturally create this interface by activating transcription of a programmable RNA sequence upon detection of a chemical target. However, there are substantial challenges in combining aTFs and TMSD circuits to function together in situ, such as interference between RNA polymerase (RNAP) and nucleic acid gates^27^, the lack of experimental characterization of RNA-mediated TMSD circuits^28^ and the complexities of RNA folding hindering TMSD circuit function.

Here, we address these challenges by developing design rules to interface the sensing layers of ROSALIND^7^ with TMSD circuits. We first show that the design of a new DNA signal gate can be optimized to enable T7 RNAP-driven in vitro transcription (IVT) and TMSD within the same reaction. Next, we develop the RNA secondary structure design rules to tune the reaction kinetics of TMSD, notably improving the response speed. We also apply this principle to interface TMSD with several different aTFs to create biosensors for their cognate ligands. We then showcase the programmability of the platform by building 12 different circuits that implement seven different logic functions (NOT, OR, AND, NOR, IMPLY, NIMPLY, NAND). Finally, using a model-driven approach, we build a multilayer TMSD circuit that acts like an analog-to-digital converter (ADC) to create a series of binary outputs that encode the concentration range of the target molecule. Taken together, this work demonstrates that TMSD can be used to implement molecular computations to expand the capabilities of cell-free biosensors.

## Results

### Engineering TMSD to be compatible with IVT

To interface ROSALIND with TMSD, we first sought to validate that a single-stranded RNA can strand-displace a DNA signal gate. We modified a DNA signal gate from a previous work to create a gate with an eight-nucleotide toehold on its 3′-end, with one strand labeled with a fluorophore and the other strand with a quencher (Supplementary Data 1)^29^. We then designed an invading RNA strand (InvadeR) to be fully complementary to the fluorophore strand so that it strand-displaces the quencher strand to generate a fluorescent output. When we combined purified InvadeR with the DNA signal gate, we observed fluorescence activation over a no InvadeR control (Supplementary Figs. 1 and 2a). In this way, InvadeR behaved similarly to an invading ssDNA strand (InvadeD), although titration of InvadeR resulted in a plateau of fluorescence at lower concentrations than InvadeD (Supplementary Fig. 2a,b). Notably, NUPACK^30^ predicts that InvadeD has a less stable structure than InvadeR, and that InvadeR can bind to itself to form a duplex, which could inhibit the function of InvadeR (Supplementary Fig. 2c–e, Supplementary Data 2 and 3).

**Extended Data Fig. 1:**
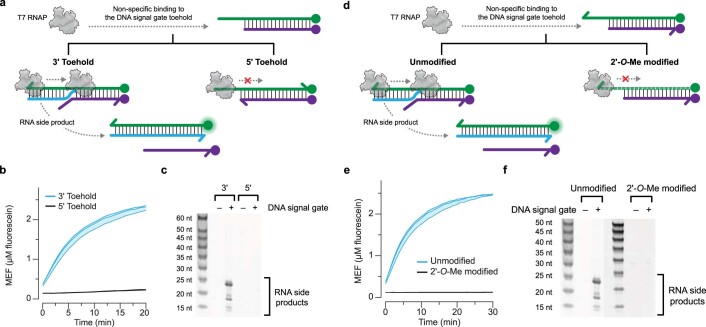
Toehold-mediated DNA strand displacement can be used to track RNA outputs with an appropriately designed DNA gate. **a**, In the presence of IVT components, T7 RNAP can non-specifically bind to the toehold region of the DNA signal gate. When the toehold is on the 3’ end of the gate, this leads to transcription of unwanted RNA side products that can displace the quencher strand. This process is blocked when the toehold is on the 5’ end of the gate. **b**, The 3’ toehold DNA signal gate leads to fluorescence activation in the presence of T7 RNAP, while the 5’ toehold DNA signal gate does not. **c**, When the reaction products from **b** were extracted and run on an urea-polyacrylamide gel, RNA side products appear only from the 3’ toehold DNA signal gate. A negative control where no DNA signal gate is present in the reaction (–) was run alongside for both 3’ and 5’ toeholds. **d**, Modifying the DNA gate with 2’-O-methyl oligonucleotides prevents promoter-independent transcription by T7 RNAP. **e**, When the 3’ toehold DNA signal gate is modified with 2’-O-methyl oligonucleotides, no fluorescence activation is observed in the absence of a T7 RNAP-driven IVT template. **f**, When the reactions from **e** were run on an urea- polyacrylamide gel, no RNA side products were observed from the 2’-O-methyl DNA signal gate. A negative control where no DNA signal gate is present in the reaction (–) was run alongside for both unmodified and modified gates. Data shown in **b** and **e** are n = 3 independent biological replicates each plotted as a line with raw fluorescence standardized to MEF (μM fluorescein). Shading indicates the average of the replicates ± standard deviation. Data shown in **c** and **f** are a representative of n = 3 independent biological replicates. The uncropped, unprocessed gel image shown in **c** and **f** is available as **Source Data**. Source data

We next determined if InvadeR can be transcribed in situ in the presence of the DNA signal gate to generate a signal. Following the ROSALIND platform design, we chose a fast, processive phage polymerase, T7 RNAP, and configured the DNA template to consist of the minimal 17-base pair (bp) T7 promoter sequence followed by two initiating guanines and the InvadeR sequence (Supplementary Data 1). We initially observed that T7 RNAP could generate a fluorescent signal from the DNA signal gate alone (Extended Data Fig. 1b). On the basis of previous reports^27,31–33^, we hypothesized that T7 RNAP was initiating transcription from the 3′ toehold region of the DNA signal gate, causing strand displacement and signal generation (Extended Data Fig. 1a). To test this hypothesis, we reversed the polarity of the DNA signal gate to include a 5′ toehold end and observed no fluorescence signal from the 5′ toehold DNA signal gate (Extended Data Fig. 1b). Urea–polyacrylamide gel electrophoresis (urea–PAGE) analysis of RNA species from each IVT reaction also showed RNA side products only from the reaction with the 3′ toehold DNA signal gate (Extended Data Fig. 1c). We confirmed that 2′-*O*-methlyation of the DNA signal gate^33^ similarly eliminates spurious transcription (Extended Data Fig. 1d–f).

### Interfacing IVT with TMSD outputs

**Fig. 2:**
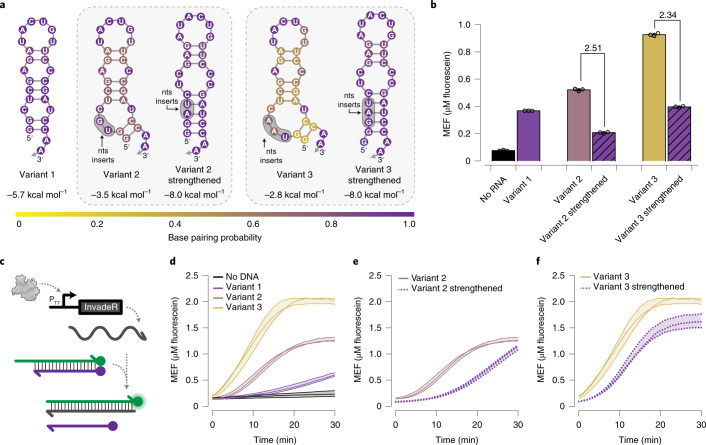
Secondary structure of InvadeR impacts TMSD efficiency. **a**, Three different variants of InvadeR were designed. Variant 1 includes the two initiating guanines followed by the sequence fully complementary to the fluorophore strand. For variants 2 and 3, two or three additional nucleotides were inserted between the initiating guanines and the InvadeR sequence (shaded regions), such that they disrupt the secondary structure at its base. Strengthened versions of variants 2 and 3 were created to stabilize the structure, while keeping the number and positions of the inserted nucleotides consistent. All variants have the same number of nucleotides that interact with the DNA signal gate. Minimum free energies and base pairing probabilities for each structure are predicted using NUPACK at 37 °C^30^. nts, nucleotides. **b**, Gel-purified InvadeR variants (5 µM) were added to an equimolar amount of the DNA signal gate, and fluorescence activation was quantified. Variant 3 generates the highest fluorescent signal followed by variants 2 and 1, whereas both strengthening mutants show a decrease in signal from their respective variants by the fold reduction indicated above the bars. **c**, When a DNA template encoding InvadeR is included with T7 RNAP and the DNA signal gate, the RNA output can be tracked in situ by monitoring fluorescence activation from the signal gate. **d**, Comparison of fluorescence kinetics of the three variants from IVT using an equimolar DNA template (50 nM) or a no-template negative control. **e**,**f**, Comparison of fluorescence kinetics between variants and their strengthening mutants for variant 2 (**e**) and variant 3 (**f**), shows that strengthening base pairs negatively impact fluorescence kinetics. All data shown are *n* = 3 independent biological replicates each plotted as a point (**b**) or a line (**d**–**f**) with raw fluorescence standardized to MEF (µM fluorescein). Each bar height in **b** represents the average over these replicates. Error bars (**b**) and shading (**d**–**f**) indicate the average of the replicates ± s.d. Source data

With the optimized DNA signal gate design, we next used TMSD to track RNA outputs generated by T7 RNAP-driven IVT in situ. We focused on optimizing the design of InvadeR for rapid signal generation. On the basis of the results in Supplementary Fig. 2, we hypothesized that the secondary structure of InvadeR would play a critical role in TMSD efficiency, for example by interfering with toehold binding and strand invasion^34,35^. To test this hypothesis, we designed five different variants of InvadeR each with varying predicted stabilities and secondary structures on the 3′-end (Fig. 2a). When an equimolar amount of each gel-purified InvadeR variant was added to the DNA signal gate, we observed that the magnitudes of fluorescence signals were ordered according to the predicted minimum free energies of each variant (Fig. 2b). Furthermore, each strengthened version showed substantially lower fluorescence than the corresponding un-strengthened variant.

We then tested the TMSD reaction kinetics of the variants transcribed in situ. When 50 nM of the DNA template encoding each InvadeR variant was added to the IVT reaction mixture (Fig. 2c), we observed the fastest fluorescence activation from variant 3 followed by variants 2 and 1 (Fig. 2d), in agreement with the previous experiment. We also observed slower responses from the strengthened versions, reaffirming our hypothesis (Fig. 2e,f).

**Extended Data Fig. 2:**
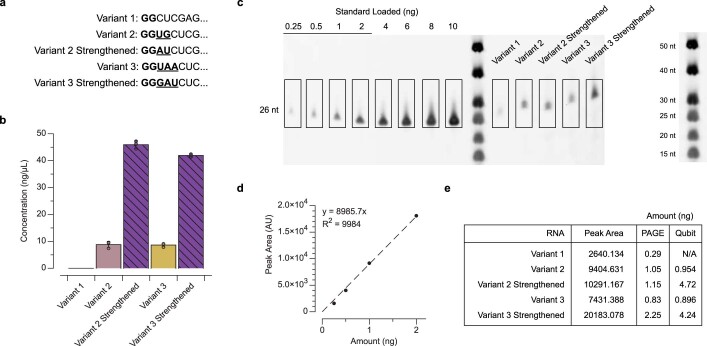
Transcription efficiency impacts the speed of TMSD. **a**, The eight initially transcribed nucleotides of each InvadeR variant in Fig. 2a. Nucleotides that are not part of the InvadeR sequences are bolded, and inserted nucleotides are underlined. **b**, Concentrations of each variant from IVT reactions measured by the Qubit RNA HS assay kit (Invitrogen, catalog no. Q32852). Each variant was produced *in situ* in the presence of the DNA signal gate for 30 min and extracted (see the RNA extraction from IVT reactions section in **Materials and Methods**). The concentration of variant 1 was too low for Qubit quantification. **c**, The samples measured in **b** were run on an urea-polyacrylamide gel and stained with SYBR gold. Titration of an RNA standard of a similar length was performed to determine the linear range of band peak area quantified by Fiji–ImageJ^56^. **d**, A calibration curve was constructed by plotting the peak area computed from Fiji–ImageJ quantification against the total amount of standard loaded. **e**, Using the calibration curve in **d**, the total amount of RNA for each variant was determined and compared to the measurements made by Qubit in **b**. Data in **b** are shown for n = 3 independent biological replicates each plotted as a point with the bar height representing the average. Error bars indicate the average of the replicates ± standard deviation. Data shown in **c** are a representative of n = 3 independent biological replicates. The uncropped, unprocessed gel image shown in **c** is available as **Source Data**. Source data

However, we observed discrepancies between the quantitative predicted secondary structure stabilities and the reaction kinetics. Specifically, the strengthened variants with lower minimum free-energy values show faster reaction kinetics and higher end-point fluorescence values than variant 1 (Fig. 2d–f), conflicting with the results observed in Fig. 2b. We discovered that varying the T7 RNAP transcription efficiency of each DNA template^36^ contributes to this discrepancy, although RNA secondary structure has a greater impact on the TMSD response speed (Extended Data Fig. 2). We also found that adding a T7 terminator sequence at the end of the DNA template speeds up the reaction, although not considerably (Supplementary Fig. 3 and Supplementary Data 3).

Together, these results show that incorporating both secondary structure and transcription efficiency considerations into InvadeR design principles can be leveraged to enhance reaction speed.

### Interfacing cell-free biosensors with TMSD outputs

**Fig. 3:**
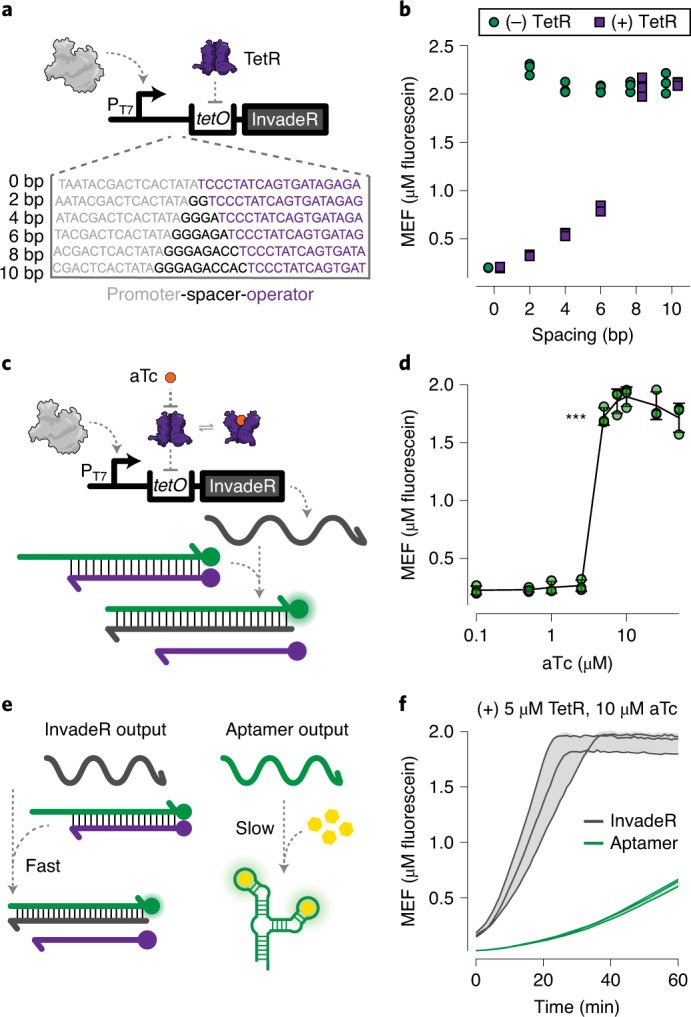
Transcription of InvadeR can be regulated by aTFs. **a**, IVTs can be allosterically regulated with a template configured to bind a purified aTF (TetR) via an operator sequence (*tetO*) placed downstream of the T7 promoter. A series of spacers at 2-bp intervals was constructed to evaluate the impact of spacer length on the ability of TetR to regulate the transcription of InvadeR. **b**, Endpoint data (at 1 h) shown for promoter–operator spacer variants regulated (with 5 μM TetR dimer, 50 nM DNA template) and unregulated (without TetR). **c**, Induction of a TetR-regulated IVT reaction occurs in the presence of the cognate ligand, aTc, which binds to TetR and prevents its binding to *tetO*. This allows transcription to proceed, leading to fluorescence activation via TMSD. **d**, Dose response with aTc, measured at 1 h with 50 nM DNA template and 5 μM TetR dimer. The lowest ligand concentration at which the signal is distinguishable from the background was determined using a two-sided, heteroscedastic Student’s *t*-test against the no-ligand condition, and the *P* value range is indicated by asterisks (***P < 0.001, **P = 0.001–0.01, *P = 0.01–0.05, the exact *P* value for 5 µM aTc = 5.064 × 10^−5^). Exact *P* values along with degrees of freedom for all ligand concentrations tested can be found in the source data. Data for the no-ligand condition were excluded because the *x* axis is on the log scale and are presented in the source data. **e**, The speed of the TMSD output is faster than that of the RNA aptamer output. **f**, Comparison of fluorescence kinetics between the TetR-regulated InvadeR and aptamer outputs when induced with 10 μM aTc. All data shown are *n* = 3 independent biological replicates each plotted as a point (**b**,**d**) or a line (**f**) with raw fluorescence values standardized to MEF (μM fluorescein). Error bars (**d**) and shading (**f**) indicate the average of the replicates ± s.d. Source data

Next, we determined whether the transcription of InvadeR can be regulated with an aTF to create a cell-free biosensor that uses TMSD outputs. This required us to insert an aTF operator sequence in between the T7 promotor and InvadeR sequence to allow aTF transcription regulation. We previously demonstrated that the spacing between the T7 promoter sequence and the aTF operator sequence is important for efficient regulation of IVT in ROSALIND reactions^7,37^. Similarly, we found that a 2-bp spacer generates a robust TMSD signal without TetR, which was reduced to nearly baseline levels when regulated by TetR (Fig. 3a,b).

Using the 2-bp spacer, we next determined whether TetR can be de-repressed with its cognate ligand, anhydrotetracycline (aTc) to allow transcription of InvadeR (Fig. 3c). When a range of aTc concentrations was added to IVT reactions each containing 50 nM DNA template, 5 µM TetR dimer and 5 µM DNA signal gate, we observed a strong repression down to low micromolar amounts of aTc, with half-maximal induction between 2.5 and 5 µM of aTc (Fig. 3d).

Owing to the rapid speed of TMSD reactions^17^, we hypothesized that the ligand-mediated induction speed of the InvadeR output would be much faster than the previously used ROSALIND RNA aptamer output that is subject to slow chromophore binding and misfolding^34,38,39^ (Fig. 3e). As expected, we observed that the InvadeR platform activates fluorescence visible in ~10 min, which is approximately five times faster than the RNA aptamer platform (Fig. 3f).

Overall, these results demonstrate that an aTF-based biosensor can be successfully interfaced with TMSD outputs, leading to immediate improvements in reaction speed.

### Optimizing InvadeR designs for different aTF families

**Extended Data Fig. 3:**
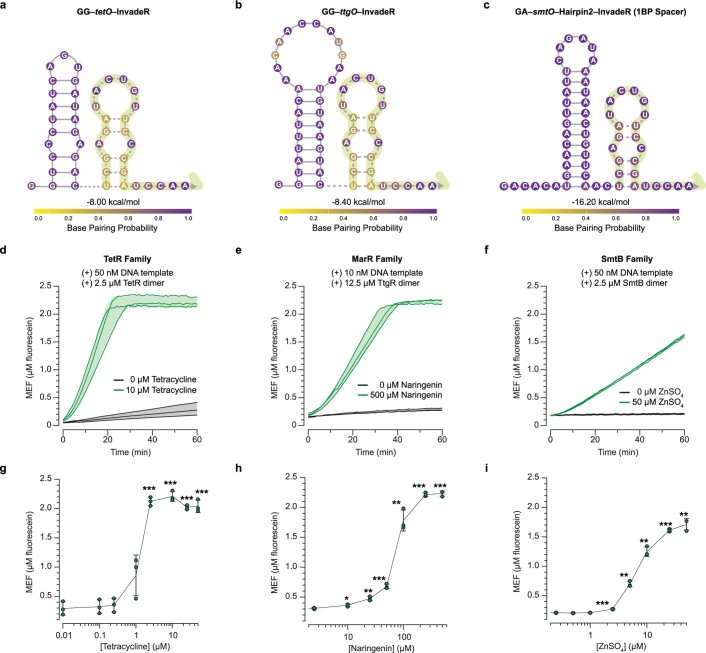
Different input molecules can be detected by modularly configuring InvadeR with an aTF operator sequence. DNA templates encoding InvadeR are modified to contain the T7 promoter followed by a 2-bp spacer and an aTF operator sequence upstream of the InvadeR sequence. Secondary structures, minimum free energies and base pairing probabilities of **a**, GG–*tetO*–InvadeR, **b**, GG–*ttgO*–InvadeR and **c**, GA–*smtO*–Hairpin2–InvadeR (1BP spacer variant) are predicted using NUPACK at 37 °C^30^. The GA–*smtO*–Hairpin2–InvadeR sequence includes a hairpin designed to minimize structural interference of InvadeR by *smtO* (Extended Data Fig. 4h, i). The sequence complementary to the signal gate is denoted with a dotted line and highlighted in green shading. **d**, TetR is used to sense tetracycline. **e**, TtgR, a MarR-family aTF, is used to sense naringenin. **f**, SmtB is used to sense zinc. Variations in activation kinetics match the trend where the predicted secondary structure of InvadeR impacts the induction speed. To evaluate the limit of detection of each sensor, a dose response curve was created for **g**, tetracycline, **h**, naringenin and **i**, ZnSO_4_ (1 h end-point data). All data shown are n = 3 independent biological replicates each plotted as a line (**d**–**f**) or a point (**g**–**i**) with raw fluorescence values standardized to MEF (μM fluorescein). Shading (**d**–**f**) and error bars (**g**–**i**) indicate the average of the replicates ± standard deviation. The ligand concentrations at which the signals are distinguishable from the background were determined using a two-sided, heteroscedastic Student’s t-test against the no-ligand condition, and their P value ranges are indicated with asterisks (***P < 0.001, **P = 0.001–0.01, *P = 0.01–0.05). Exact P values along with degrees of freedom can be found in **Source Data**. Data for the no ligand condition were excluded because the x-axis is on the log scale and are presented in **Source Data**. Source data

**Extended Data Fig. 4:**
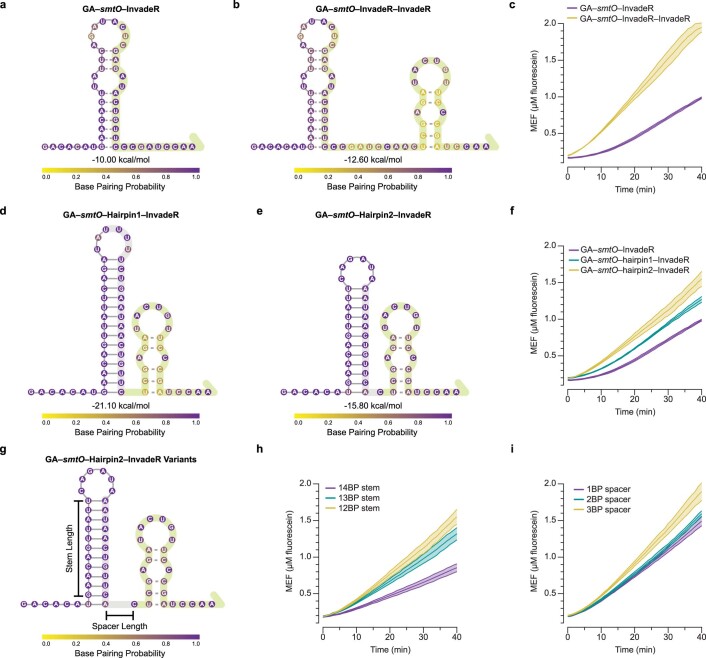
Extra nucleotides can be added between the operator and InvadeR sequence to tune the kinetics of the zinc sensor. **a**, Secondary structure, minimum free energy and base pairing probabilities of GA-*smtO*–InvadeR predicted by NUPACK at 37 °C^30^. A part of the wild type *smtO* sequence forms a strong predicted stem-loop with InvadeR. The nucleotides highlighted in green correspond to the InvadeR sequence that strand-displaces the DNA signal gate. **b**, Secondary structure, minimum free energy and base pairing probabilities of GA-*smtO*–InvadeR–InvadeR predicted by NUPACK at 37 °C^30^. **c**, Comparison of the kinetics of the unregulated GA-*smtO*–InvadeR reaction and the unregulated GA-*smtO*–InvadeR–InvadeR reaction. **d**, **e**, NUPACK-predicted secondary structures, minimum free energies and base pairing probabilities of the two GA-*smtO*–Hairpin–InvadeR variants designed to sequester the *smtO* sequence and prevent it from binding to InvadeR. The nucleotides highlighted in grey indicate the added sequestering sequence. **f**, Comparison of the kinetics of the unregulated reactions of the variants shown in **d** and **e**. **g**, The GA-*smtO*–Hairpin2–InvadeR variants were built by lengthening either the stem length or the spacer between the hairpin and the InvadeR sequence. The stem length variants are built with the 0-nt spacer, and the spacer variants are built with the 12-bp stem length. **h**, **i**, Comparison of the kinetics of the unregulated reactions of the GA-*smtO*–Hairpin2–InvadeR variants. All data shown are n = 3 independent biological replicates each plotted as a line with raw fluorescence values standardized to MEF (μM fluorescein). Shadings indicate the average of the replicates ± standard deviation. Source data

We next determined whether the system is compatible with different families of aTFs to detect various classes of small molecules. In addition to TetR, we chose TtgR^40^ and SmtB^41^ as representative aTFs of the MarR family^42^ and SmtB/ArsR family^43^, respectively. We placed the cognate operator sequence of each aTF 2-bp downstream of the T7 promoter and immediately upstream of the InvadeR sequences (Extended Data Fig. 3a–c). When tetracycline was used to induce TetR-regulated reactions, we observed a strong and robust fluorescent signal visible in ~10 min (Extended Data Fig. 3d,g). Similarly, when naringenin, a cognate ligand of TtgR, was added to TtgR-regulated reactions, we again saw robust fluorescence activation only in the presence of naringenin (Extended Data Fig. 3e,h). However, introducing the *smtO* sequence resulted in much slower reaction speeds even in unregulated reactions (Extended Data Fig. 4c). Interestingly, the *smtO* sequence is predicted to form a strong hairpin with the InvadeR sequence (Extended Data Fig. 4a), which on the basis of our results in Fig. 2 could impact reaction speeds. To improve the reaction kinetics, we designed an RNA structural insulation module to prevent the intramolecular *smtO*:InvadeR folding (Extended Data Fig. 4), which showed faster signal activation of a SmtB-regulated reaction in the presence of ZnSO_4_ with low background signal (Extended Data Fig. 3f,i).

Together, these results demonstrate that RNA design strategies can be used to modularly extend the ROSALIND platform with TMSD circuits.

### Performing logic computation with cascaded TMSD circuits

The interface of cell-free biosensors with TMSD provides opportunities to engineer modular devices that can perform programmed tasks in response to small molecule inputs. This is especially true because TMSD circuits have simpler design rules than protein circuits^44^, there exist computational models that accurately predict their behavior^34,35^ and there are an emerging set of TMSD circuit design tools^45,46^. We therefore leveraged these features to create a TMSD information processing layer for ROSALIND.

**Fig. 4:**
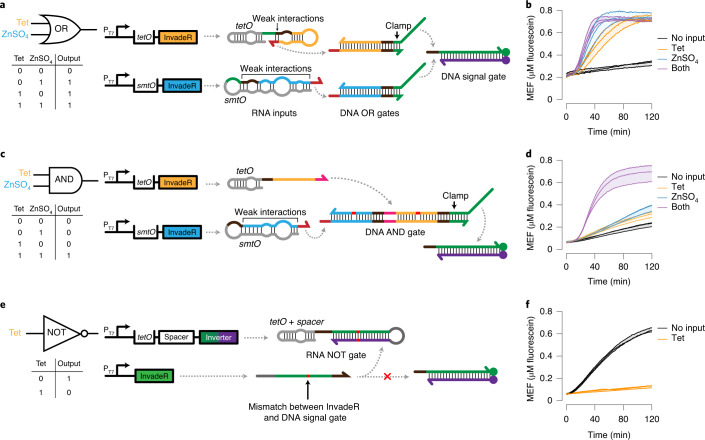
Additional gates can be layered to perform logic computation. **a**, A two-input OR gate includes two additional DNA OR gates. When transcription is activated by either ligand, InvadeR strands can react with their respective DNA OR gates to produce an output strand with a domain (green) that can invade the DNA signal gate. **b**, When the two-input OR gate is activated by either tetracycline, ZnSO_4_ or both, fluorescence activation is observed. **c**, A two-input AND gate contains a DNA AND gate that requires both InvadeR strands to dissociate the output strand to reveal the toehold complementary to the signal gate. Design features such as thermodynamic drivers (highlighted in red) and a clamp domain are implemented to facilitate efficient TMSD with minimal leak (Extended Data Fig. 6). **d**, The two-input AND gate activates fluorescence only when both tetracycline and ZnSO_4_ are present. **e**, A NOT gate includes two DNA templates: an unregulated InvadeR template and an aTF-regulated inverter template. Upon transcription, the aTF-regulated inverter forms a hairpin structure that resembles the DNA signal gate to create an RNA NOT gate. Unregulated InvadeR preferably reacts with the RNA NOT gate because of a greater number of base pair interactions and a base pair mismatch with the DNA signal gate (red). A spacer sequence is included to prevent the *tetO* sequence from interfering with the RNA NOT gate. **f**, When implemented with a tetracycline sensor, the NOT gate generates signal in the absence of tetracycline. All data shown are *n* = 3 independent biological replicates each plotted as a line with raw fluorescence value standardized to MEF (μM fluorescein). Shading indicates the average of the replicates ± s.d. Domains with the same color share the same sequence except for the AND gate where the domain highlighted in orange is modified from the orange domain in the OR gate to improve its TMSD efficiency. All nucleic acid gates are drawn according to the secondary structures predicted using NUPACK at 37 °C^30^. The sequence of each domain and the concentrations of components in each gate can be found in Supplementary Data 4 and 5, respectively. Source data

**Extended Data Fig. 5:**
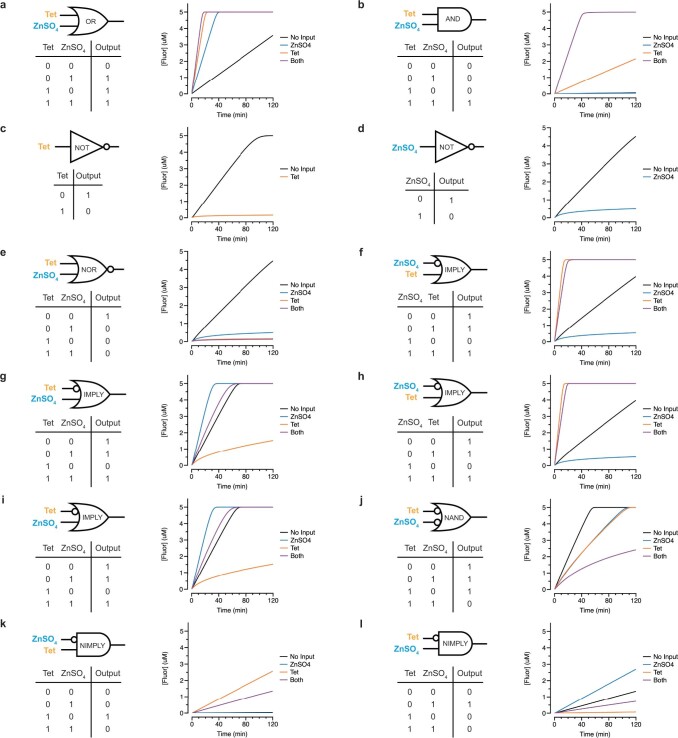
ODE Modeling of Logic Gates. Simulations of logic gates discussed in Figs. 4, 5 and Extended Data Fig. 7 are shown along with the expected simulated trajectory trends. All Jupyter notebook codes used to simulate the results are available as Supplementary Data 6, and the method used to develop the ODE model for each representative logic gate is discussed in the Supplementary Information. Source data

We first designed and built logic gates to process two different ligands as inputs to the system. Specifically, we adapted previous designs of TMSD logic gates, AND and OR, that detect nucleic acid inputs^12,14,15,46^ (Fig. 4). We implemented OR logic by designing DNA OR gates that act as an intermediate layer between InvadeR and the signal gate. Once the transcription of InvadeR is triggered by a chemical ligand, InvadeR performs TMSD on its corresponding DNA OR gate to release a ssDNA output strand, which can subsequently invade the DNA signal gate to produce a signal (Fig. 4a and Supplementary Data 4)^46^. The two DNA OR gates share the same output domain (green), but are activated by InvadeR regulated by two different aTFs. Including these OR gates alongside DNA templates, aTFs and the signal gate led to fast signal generation except when no target ligands were present (Fig. 4b and Supplementary Data 5). Modeling the OR gate using a set of ordinary differential equations (ODEs) that describe the IVT and TMSD reactions (see Supplementary Information for details on the ODE model used) matched the experimental trends (Extended Data Fig. 5a and Supplementary Data 6).

**Extended Data Fig. 6:**
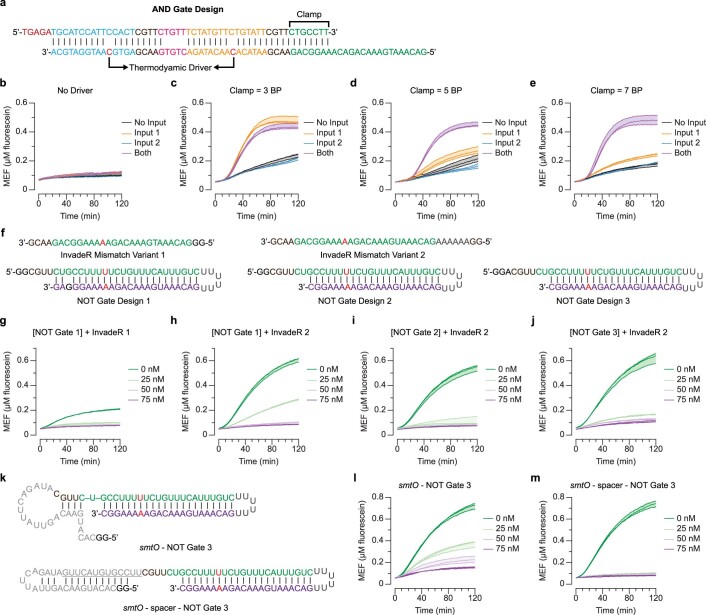
Design Features of Basic Logic Gate Components. **a**, The sequence and design of the DNA AND gate shown in Fig. 4c. The mismatches act as the thermodynamic driver to run the reaction forward. The clamp domain prevents the top strand from being strand-displaced only with Input 1 (orange). **b**, Without thermodynamic drivers, no signal is observed. Lengthening the clamp reduces the leak by Input 1. The leak observed from a **c**, 3-bp, **d**, 5-bp and **e**, 7-bp clamp. **f**, The sequences and designs of InvadeR and RNA NOT gates without any operator sequences. The mismatch between InvadeR and the signal gate is highlighted in red. Variant 2 incorporates 6 extra adenines that strengthen its interactions with the NOT gate. NOT gate 1 has a 4-nt toehold while NOT gate 2 and 3 have 6-nt toeholds. NOT gate 3 has an extra adenine following the initiating guanines that increases its transcription efficiency^36^. Titration of the template encoding NOT gate 1 in the presence of 25 nM of the template encoding InvadeR **g**, variant 1 and **h**, variant 2. While variant 2 requires more of the NOT gate template to block signal, stronger signal is observed from variant 2 without the NOT gate. Titration of the template encoding NOT gate **i**, 2 and **j**, 3 in the presence of 25 nM of the InvadeR variant 2 template. NOT gate 2 and 3 are more efficient at sequestering InvadeR than NOT gate 1. **k**, Incorporating the operator sequence into the NOT gate impacts its structure. NUPACK predicts that the *smtO* sequence (light grey) interacts with a part of NOT gate 3, blocking the toehold^30^. A spacer sequence (underlined) can restore the expected structure of the NOT gate. Titration of the template encoding **l**, *smtO*–NOT gate 3 and **k**, *smtO*–spacer–NOT gate 3 in the presence of 25 nM of the InvadeR variant 2 template. All reactions shown are unregulated. All data shown are n = 3 independent biological replicates each plotted as a line with raw fluorescence standardized to MEF (µM fluorescein). Shading indicates the average of the replicates ± standard deviation. Source data

Next, we designed an RNA-based TMSD AND gate by adapting a previous AND gate design^47^ that consists of three domains: domain 1 complementary to InvadeR 1 (blue), domain 2 complementary to InvadeR 2 (orange) and domain 3 complementary to the DNA signal gate (green) (Fig. 4c and Supplementary Data 5). InvadeR strands were designed to have different toehold sequences to minimize unwanted toehold binding to the incorrect AND gate domain. Implementation of this architecture in sensing reactions using TetR and SmtB initially led to no fluorescence activation in any condition (Extended Data Fig. 6b). To drive TMSD reactions forward, we incorporated mismatches in the AND gate that act as TMSD thermodynamic drivers^48^ (Extended Data Fig. 6a). Although the thermodynamic drivers showed signal activation by InvadeR, substantial leak was observed with the InvadeR 1 only condition (Extended Data Fig. 6c). To reduce this leak, we implemented an additional design feature called a clamp in domain 3 of the AND gate which prevents partial TMSD in the presence of InvadeR 1 only^49^. By increasing the clamp length to 7 bp, we built an AND gate that requires both tetracycline and ZnSO_4_ inducible InvadeR strands for signal generation (Fig. 4d, Extended Data Figs. 5b and 6e and Supplementary Data 5).

**Extended Data Fig. 7:**
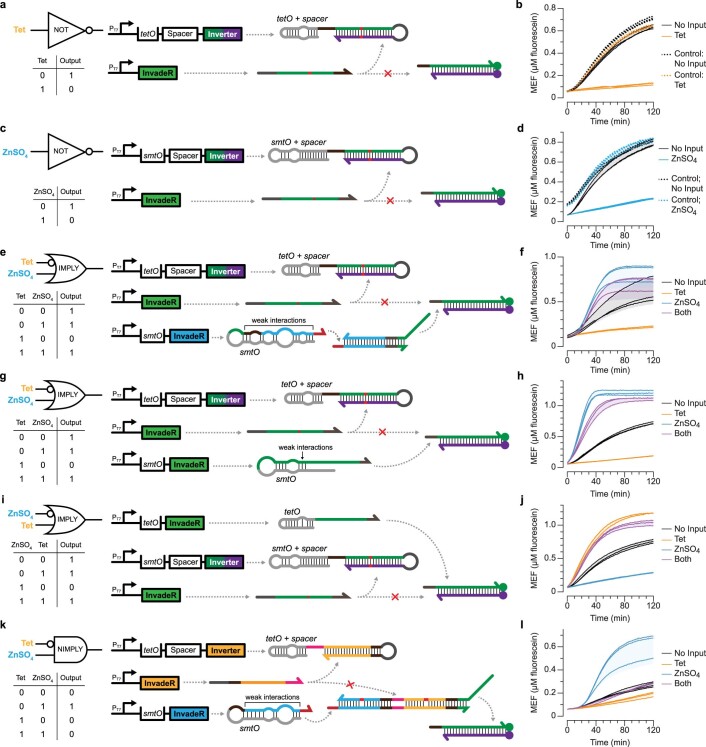
Logic gates can be built modularly using ligand-induced RNA inputs. **a**, Tetracycline–induced NOT gate shown in Fig. 4e. **b**, A template encoding the shuffled sequence of the TetR-regulated NOT gate was used as a control to demonstrate that the tetracycline-induced signal reduction is not due to resource limitations. **c**, ZnSO_4_–induced NOT gate can be designed the same way. The spacer sequence was added to prevent the *smtO* sequence from disrupting the NOT gate structure (Extended Data Fig. 6). **d**, In the presence of ZnSO_4_, the fluorescence signal is deactivated. Similarly, control reactions with a template encoding the shuffled sequence of the SmtB-regulated NOT gate are shown. **e**, A tet IMPLY ZnSO_4_ gate designed as described in Fig. 5c. **f**, Fluorescence activation is observed unless only tetracycline is added. **g**, A tet IMPLY ZnSO_4_ gate can be alternatively built by including a ZnSO_4_–inducible template that interacts with the signal gate instead of the OR gate. **h**, The alternative IMPLY gate performs the expected logic computation. A faster signal generation from the ZnSO_4_-induced conditions is observed since the ZnSO_4_-induced RNA strands directly perform TMSD on the signal gate. **i**, The alternative approach to building the IMPLY gate can be applied to the ZnSO_4_ IMPLY tet gate, and **j**, the gate performs the expected logic computation. **k**, A tet NIMPLY ZnSO_4_ gate is designed the same way as the ZnSO_4_ NIMPLY tet gate shown in Fig. 5g. **l**, The tet NIMPLY ZnSO_4_ gate performs the expected logic computation. All the experimental conditions used in this figure can be found in Supplementary Data 5. All data shown are n = 3 independent biological replicates each plotted as a line with raw fluorescence standardized to MEF (µM fluorescein). Shading indicates the average of the replicates ± standard deviation. Source data

Building more complex logic gates beyond AND and OR requires a NOT circuit, which blocks signal in the presence of a target ligand. To achieve signal inversion, we designed an RNA NOT gate that is capable of sequestering InvadeR away from the DNA signal gate (Fig. 4e and Supplementary Data 4)^47,50^. To bias InvadeR binding to the RNA NOT gate, we included three design features: (1) a longer toehold on the RNA NOT gate than on the DNA signal gate; (2) additional base pairing in the loop of the RNA NOT gate; and (3) a mismatch (highlighted in red) between InvadeR and the DNA signal gate (Extended Data Fig. 6f–j). We also designed a spacer sequence between the RNA NOT gate and the *tetO* operator to structurally insulate the *tetO* sequence from the NOT gate sequence (Fig. 4e). When these design features were implemented, we observed signal reduction in the presence of tetracycline (Fig. 4f, Extended Data Fig. 5c and Supplementary Data 5). No signal inversion was observed from a control template whose sequence is shuffled from the tetracycline-inducible RNA NOT gate (Extended Data Fig. 7a,b). The same design architecture was applied to build a ZnSO_4_-inducible RNA NOT gate (Extended Data Figs. 5d, 6k–m and 7c,d, and Supplementary Data 4 and 5).

These results establish a set of design rules for building cascaded TMSD circuits for more complex logic gate computation.

### Layering gates to perform complex logic computations

We next layered the basic logic components to perform complex logic computation including NOR, NAND, IMPLY and NIMPLY.

**Fig. 5:**
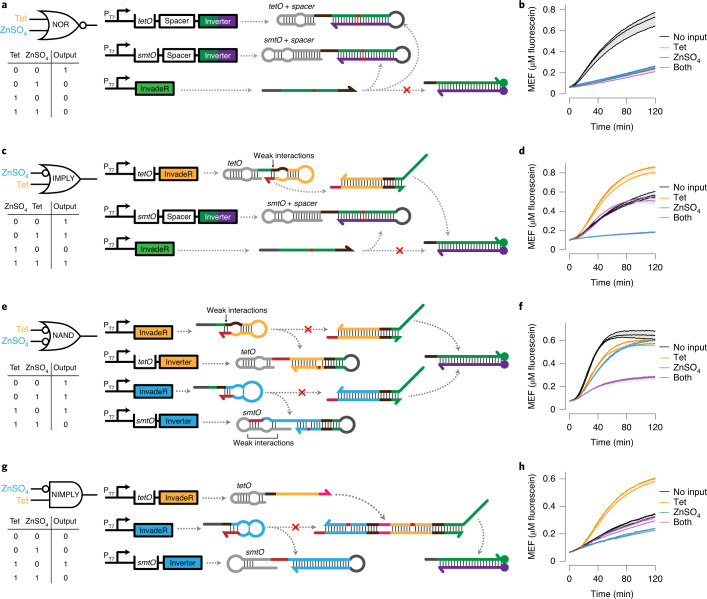
Logic component layering allows more complex computation. **a**, A two-input NOR gate is built by layering two RNA NOT gates. The NOT gates are regulated by either TetR or SmtB that sequester the unregulated InvadeR molecules from the DNA signal gate. **b**, Fluorescence activation is observed only in the absence of both inputs. **c**, A two-input IMPLY gate combines a DNA OR gate with an RNA NOT gate. In this specific example (ZnSO_4_ IMPLY tetracycline), the OR gate is regulated by TetR, and the NOT gate is regulated by SmtB, preventing signal generation in the presence of ZnSO_4_ only. **d**, Fluorescence activation is observed unless only ZnSO_4_ is added. Faster signal generation is observed from the tetracycline only input condition due to no mismatch between the OR gate output strand and the signal gate. **e**, A two-input NAND gate layers two unregulated DNA OR gates with two regulated RNA NOT gates. In this configuration, the presence of both inputs is required to hinder signal generation. Thermodynamic drivers (highlighted in red) are incorporated in the NOT gates to favor the interactions with their respective InvadeR strands. **f**, The expected NAND gate computation is observed. **g**, A two-input NIMPLY gate is built by combining the DNA AND gate and the RNA NOT gate. In this specific example, tetracycline-induced InvadeR and unregulated InvadeR are required for the AND gate activation. A SmtB-regulated NOT gate sequesters the unregulated InvadeR. **h**, Fluorescence activation is observed in the presence of tetracycline only. All data shown are *n* = 3 independent biological replicates each plotted as a line with raw fluorescence value standardized to MEF (μM fluorescein). Shading indicates the average of the replicates ± s.d. Domains with the same color share the same sequence except for the AND gate where the domain highlighted in orange is modified from the orange domain in the OR gate. All nucleic acid gates are drawn according to the secondary structures predicted using NUPACK at 37 °C^30^. The sequence of each domain and the concentrations of components in each gate can be found in Supplementary Data 4 and 5, respectively. Source data

We began with NOR—an inversion of the OR gate that generates signal only when all inputs are absent—by combining two RNA NOT gates each regulated by TetR or SmtB (Fig. 5a and Supplementary Data 4). With both RNA NOT gates designed to sequester the same constitutively expressed InvadeR, we observed fluorescence activation only in the absence of both ligand inputs (Fig. 5b, Extended Data Fig. 5e and Supplementary Data 5).

Next, we focused on the A IMPLY B architecture, which blocks signal only when A is present and B is absent. The ZnSO_4_ IMPLY tetracycline gate was built by layering the tetracycline-induced DNA OR gate with the ZnSO_4_-induced RNA NOT gate (Fig. 5c and Supplementary Data 4). When implemented, the gate generated signal in all input conditions except when only ZnSO_4_ is present (Fig. 5d and Extended Data Fig. 5f). We note, however, that the built-in mismatch between InvadeR and the signal gate (highlighted in red) resulted in a slower and lower fluorescent signal from the no input or both input condition than from the tetracycline only condition, and similar effects were observed from the tetracycline IMPLY ZnSO_4_ gate (Extended Data Figs. 5g and 7e,f). Interestingly, IMPLY gates can also be built without the DNA OR gate, which allows direct interactions between the ligand-induced InvadeR strand and the signal gate (Extended Data Figs. 5h,i and 7g–j). The ability to design alternative gate architectures for the same logic computation highlights the platform’s modularity.

We then constructed a NAND gate, which combines NOT and AND gates to produce signals in all conditions except when both inputs are present. We explored two design options: (1) inversion of an AND gate output strand (A NAND B = NOT (A AND B)); and (2) two RNA NOT gates integrated into the OR gate architecture (NOT (A AND B) = NOT A OR NOT B). Because of the sequence constraints imposed by the first design option, we chose to build the NAND gate using the second option (Fig. 5e). This design involves four different DNA templates—two unregulated templates each encoding InvadeR for its corresponding DNA OR gate and two regulated templates each encoding the RNA NOT gate capable of sequestering its respective InvadeR. To avoid the slower TMSD reaction kinetics observed in the IMPLY gate design, we instead introduced a thermodynamic driver in the RNA NOT gate (highlighted in red) to favor TMSD of InvadeR with the RNA NOT gate over that with the DNA OR gate (Fig. 5e). When implemented, both tetracycline and ZnSO_4_ were required to prevent signal generation (Fig. 5f and Extended Data. Fig. 5j).

Finally, we designed the A NIMPLY B gate, which combines AND and NOT gates to produce an output only when input A is present alone. The specific NIMPLY gate design shown in Fig. 5g uses the ZnSO_4_-induced RNA NOT gate alongside a DNA AND gate that requires both unregulated InvadeR and tetracycline-induced InvadeR for activation. Both the ZnSO_4_ NIMPLY tetracycline gate as well as the tetracycline NIMPLY ZnSO_4_ gate performed the expected logic gate computations (Fig. 5h and Extended Data Figs. 5k,l and 7k).

Together, these results show that basic logic gate components can be layered to perform more complex molecular computation using small molecules as inputs to the system.

### Using a TMSD circuit to quantify biosensor outputs

To demonstrate a practical application of TMSD information processing, we next focused on quantifying biosensor outputs. In typical cell-free biosensing systems, outputs are generated when the input compound concentration is above a detection threshold, creating a ‘presence/absence’ results interpretation tuned to this threshold^51^. This detection threshold is determined by the aTF–ligand and aTF–DNA binding constants, which can be difficult to tune. To address this limitation, we designed a system like an ADC circuit^52^ to create a series of binary outputs that encode the analog input concentration of the target compound (Supplementary Fig. 4).

**Fig. 6:**
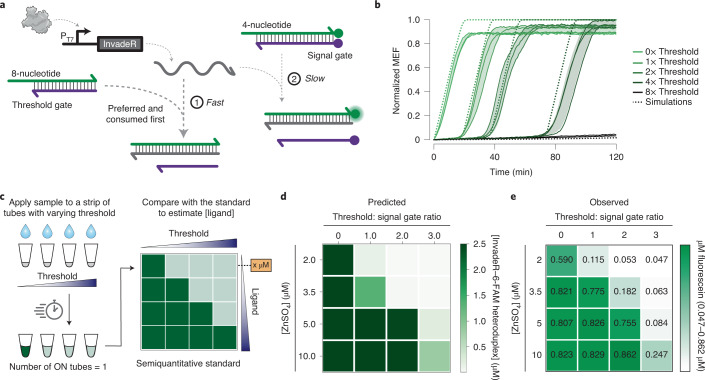
Quantifying ligand concentration with a genetic ADC circuit. **a**, Increasing the length of the DNA gate toehold region can be used to speed the strand invasion process. An unlabeled DNA gate with a longer toehold (eight nucleotides) can then preferentially react with InvadeR, acting as a programmable threshold. InvadeR can only strand-displace the signal gate (four-nucleotide toehold) after the threshold gate is exhausted. **b**, Titrating the eight-nucleotide toehold threshold gate in different ratios above a fixed signal gate concentration (0–8×) results in a time delay in fluorescence activation that can be quantitatively modeled with ODE simulations (dotted lines). All data shown for *n* = 3 independent biological replicates each plotted as a line. Raw fluorescence values were first standardized to MEF (μM fluorescein) and normalized to the maximum MEF among all conditions to accommodate their comparison with the simulations (See Methods for the normalization method used). Shading indicates the average of the replicates ± s.d. **c**, A molecular ADC circuit is made by constructing a strip of tests of the same sensor, with each test containing a different concentration of the DNA threshold gate. A higher threshold gate concentration requires a higher ligand concentration to activate fluorescence. When the same sample is applied to each tube, a user can obtain semiquantitative information about the concentration of ligand present in the sample (analog input) by identifying the series of tubes that activate (binary digital output). Here, we define a tube with its MEF value >0.5 as ‘ON’ because the visible threshold is around the indicated value. **d**,**e**, Characterization of a molecular ADC circuit for zinc using ODE simulations (**d**) and endpoint experimental data at 100 min (**e**) generated using the SmtB-regulated zinc sensor. The values on the heatmap represent the average MEF (μM fluorescein) of *n* = 3 independent biological replicates (see Extended Data Fig. 8 for all data). Source data

To construct a genetic ADC circuit, we first created a comparator circuit—a building block of ADCs that produces a ‘True’ binary output when the input is above a predefined threshold. Thresholding is possible in TMSD because the reaction kinetics can be precisely increased by lengthening DNA gate toehold regions^17^ (Fig. 6a). Using this feature, we built an unlabeled DNA threshold gate that shares the same sequence with the DNA signal gate but with a longer eight-nucleotide toehold (Fig. 6a). Because the signal should be activated only after the threshold gate is completely consumed, we reasoned that by tuning the amount of the threshold gate, we can precisely control the time at which InvadeR activates fluorescence from the signal gate. Modeling this kinetic behavior showed that this is indeed the predicted behavior of the setup, and we experimentally observed quantitative agreement with modeling predictions (Fig. 6b). In this way, a thresholded TMSD reaction acts as a ‘kinetic’ comparator circuit—for a given input, the time at which signal generation occurs is proportional to the amount of the threshold gate.

**Extended Data Fig. 8:**
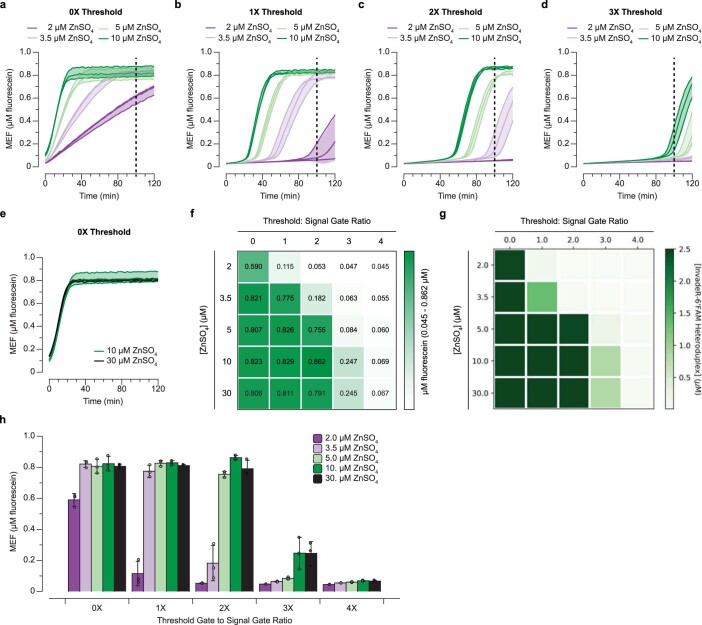
The Semi-quantitative Standard Generated by the Genetic ADC Circuit Built with the Zinc Sensor. The kinetic traces corresponding to the data shown in Fig. 6e are presented for the 8-nt threshold gate in different ratios above a fixed signal gate concentration (**a**, 0X, **b**, 1X, **c**, 2X and **d**, 3X threshold). The time point (t = 100 min) at which the heatmap was created is indicated with a vertical dotted line. The differences in the response speed for different zinc concentrations are essential for creating the standard. **e**, The 10 μM and 30 μM zinc conditions show no kinetic differences without any threshold gate. **f**, **g**, The functional characterization and ODE predictions of the ADC circuit at 100 min from Fig. 6e,d, respectively, including the 30 μM zinc and 4X threshold conditions. The 10 μM and 30 μM zinc conditions behave identically as predicted by the ODE model due to their identical kinetic behavior observed in **e**. **h**, The corresponding bar graph data of the semi-quantitative standard shown in **f**. All data shown are n = 3 independent biological replicates each plotted as a line (**a**–**e**) or a point (**h**) with raw fluorescence values standardized to MEF (μM fluorescein). The bars in **h** and the values on heatmap in **f** represent averages of the replicates. Shadings in **a**–**e** and error bars in **h** indicate the average of the replicates ± standard deviation. Source data

Next, we created a series of biosensing TMSD comparator circuits to act as an ADC for ligand concentration by preparing a series of reactions in which each tube contains a different amount of the threshold gate. By adding the same ligand concentration to each tube, a user can observe which tubes in the series are activated at a specific time point and obtain semiquantitative information about ligand concentration (Fig. 6c). We tested the feasibility of the approach using the same set of ODEs used in Fig. 6b with the addition of aTF–DNA and aTF–ligand binding kinetics, focusing on zinc sensing because of its relevance in municipal water supplies^53^. Using modeling simulations, we determined the threshold gate concentrations needed to activate one, two, three or four tubes after 100 min corresponding to zinc concentrations of 2, 3.5, 5 and 10 µM, respectively (Fig. 6d). We then built the corresponding genetic ADC circuit with the SmtB-regulated TMSD reactions and saw the expected pattern of signals after 100 min, where the number of activated tubes in the series increased with higher input ZnSO_4_ concentrations (Fig. 6e and Extended Data Fig. 8a–d). This implementation allows a user to determine the input zinc concentration range by directly reading out the sequence of activated tubes.

We believe that this demonstration represents the potential for TMSD circuits to act as an information processing layer of cell-free biosensors that increase ease of interpretation and the information content of the output signals.

## Discussion

**Extended Data Fig. 9:**
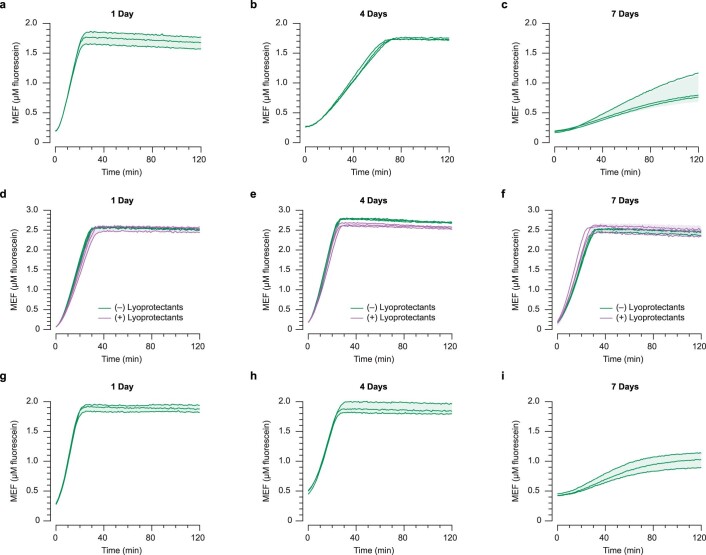
ROSALIND with TMSD can be freeze-dried. Unregulated reactions were lyophilized overnight with the addition of 50 mM sucrose and 250 mM D-mannitol as the lyoprotectants unless otherwise indicated. The lyophilized reactions were then vacuum-packaged in a light protective bag with a dri-card and kept in a cool, shaded area until usage (see **Materials and Methods** for the detailed protocol). Kinetic traces of rehydrated reactions after **a**, 1 day, **b**, 4 days and **c**, 7 days of storage are shown. There is a decrease in overall signal as well as in the response speed over time. To investigate the cause of the signal loss over time, the DNA signal gate alone was lyophilized overnight with or without the lyoprotectants, packaged and stored as described above. The DNA signal gate was rehydrated with the rest of the IVT components after **d**, 1 day, **e**, 4 days and **f**, 7 days. The response speed as well as the magnitude of the signal are maintained, indicating that the signal loss is likely due to instability of certain IVT components. To test this hypothesis, unregulated reactions with Tris-buffered NTPs, instead of NaOH-buffered NTPs, were lyophilized with the lyoprotectants, packaged and stored as described above. Kinetic traces of rehydrated reactions after **g**, 1 day, **h**, 4 days and **i**, 7 days of storage are shown. The signal loss is somewhat mitigated with Tris-buffered NTPs, but a similar degree of signal loss is observed for a long-term storage of lyophilized reactions. All data shown are n = 3 independent biological replicates each plotted as a line with raw fluorescence values standardized to MEF (μM fluorescein). Shadings indicate the average of the replicates ± standard deviation. Source data

**Extended Data Fig. 10:**
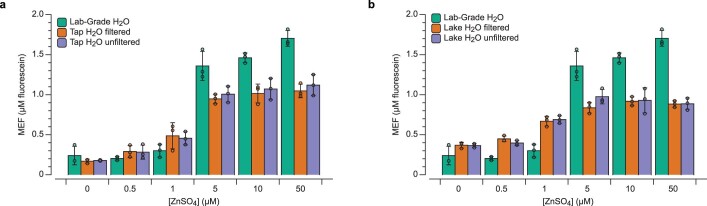
Freeze-dried ROSALIND with TMSD can be rehydrated with real-world water matrices. Zinc-sensing ROSALIND with TMSD reactions that use *smtO*–InvadeR–InvadeR shown in Extended Data Fig. 4b were freeze-dried and rehydrated with **a**, tap water and **b**, Lake Michigan water spiked with a range of concentrations of ZnSO_4_. The reactions were then incubated at 37 °C for 1 hour and characterized for fluorescence. For each water sample type, the signal was compared to that of the reactions rehydrated with laboratory-grade water spiked with the same amount of ZnSO_4_. In each case, the reactions behaved as expected and saw no difference in signal between filtered and unfiltered water samples. However, a slight signal reduction is observed from the real-world water samples compared to the ones rehydrated with the ZnSO_4_ spiked laboratory-grade water likely due to matrix effects. All data shown are n = 3 independent biological replicates each plotted as a point with raw fluorescence values standardized to MEF (μM fluorescein). Each bar height represents the average of the replicates, and error bars indicate the average of the replicates ± standard deviation. Source data

In this study, we show that TMSD circuits can be interfaced with IVT to act as an information processing layer for cell-free biosensors. The speed of TMSD outputs led to a substantial enhancement of signal generation speed, with less than 10 min time-to-detection (Fig. 3f). More importantly, we found that the simple and defined nature of ROSALIND, combined with the computational power of TMSD enabled us to layer multiple RNA–DNA gates to build 12 different circuits that implement seven different logic functions in a modular fashion (Figs. 4 and 5). Harnessing this high programmability of the platform, we also designed and validated a circuit that can estimate the concentration range of an unknown target compound within a sample (Fig. 6). Finally, this platform is amenable to lyophilization (Extended Data Fig. 9) and can function with unprocessed real-world sample matrices (Extended Data Fig. 10).

The system presented a number of design challenges including the incompatibility of 3′ toeholds in DNA gates with T7 RNAP-driven IVT reactions (Extended Data Fig. 1)^45^, interfering RNA secondary structures that can slow TMSD (Fig. 2 and Extended Data Fig. 4) and varying T7 transcription efficiency of different DNA templates^36^ (Extended Data Fig. 2). These challenges provided opportunities to develop RNA-based TMSD design principles that we believe are generalizable to other nucleic acid engineering systems (Supplementary Fig. 5).

One of the major limitations of the platform is its cost. Chemically modified oligos with purification can cost ~100 USD or more, although a single batch can be used to make hundreds of reactions. Furthermore, DNA gates often need to be gel-purified after hybridization to eliminate any unbound ssDNA strands, which can be time-intensive and laborious. We note, however, that the cost of chemical dyes for fluorescence-activating RNA aptamer reporting systems is not negligible, and the advantages provided by the TMSD system such as the improved response speed and computational power outcompete its limitations.

The key feature of this study was demonstrating the potential of TMSD circuits to expand the function of cell-free biosensors by acting as information processing layers. While an approach was recently developed to interface aTF-based biosensing with TMSD through endonuclease-mediated TMSD cascades^54^, no programmable molecular computation beyond simple contaminant detection was presented. As a demonstration, we modeled, designed and validated several layered RNA-based TMSD circuits capable of performing complex logic gate computation with small molecule inputs by adapting several elements of DNA-based TMSD logic gates (Figs. 4 and 5, and Extended Data Figs. 5–7). Although not shown, more complex logic circuits such as XOR and XNOR could be designed by layering additional gates.

To further highlight the platform’s capability for information processing, we developed a genetic ADC circuit that can be used to estimate an input ligand concentration at a semiquantitative level (Fig. 6). In particular, this circuit uses thresholding computation to convert an analog signal of an input molecule concentration into a digital output of the number of activated tubes. This development was enabled by the ability to precisely tune reaction rates of TMSD on the basis of the toehold length. We note, however, this genetic ADC circuit is different from an electrical ADC circuit in that its result depends on time of activation and not activation level, because the circuit relies on thresholding reaction kinetics rather than strictly input concentrations. As a result, this strategy is best suited to distinguishing between ligand concentrations that cause differences in output kinetics (Extended Data Fig. 8e–g).

We believe that this platform opens the door to other types of molecular computation in cell-free systems. For example, an amplification circuit such as a catalytic hairpin assembly^55^ could be applied to ROSALIND with TMSD for amplifying signals and making a sensor ultrasensitive. Beyond thresholding, other operations demonstrated in DNA seesaw gate architectures could be ported to this platform for various computations^46^. Specifically, logic gates can be extended to develop a general strategy to fix aTF ligand promiscuity^7^. In addition, because virtually any aTF that functions in an in vitro context can be used^7^, multiple DNA gates with different reporters could be added for multiplexing. The fundamental role that ADC circuits play in interfacing analog and digital electronic circuitry also holds promise for adopting additional electronic circuit designs to biochemical reactions.

Together, these results show that establishing an interface between small molecule biosensing and TMSD circuits is a promising first step towards creating a general molecular computation platform to enhance and expand the function of cell-free biosensing technologies.

## Methods

### Strains and growth medium

*Escherichia coli* strain K12 (NEB Turbo Competent *E. coli*, New England Biolabs, catalog no. C2984) was used for routine cloning. *E. coli* strain Rosetta 2(DE3)pLysS (Novagen, catalog no. 71401) was used for recombinant protein expression. Luria broth supplemented with the appropriate antibiotic(s) (100 µg ml^−1^ carbenicillin, 100 µg ml^−1^ kanamycin and/or 34 µg ml^−1^ chloramphenicol) was used as the growth media.

### DNA gate preparation

DNA signal gates used in this study were synthesized by Integrated DNA technologies as modified oligos. They were generated by denaturing a 6-carboxyfluorescein (6-FAM) modified oligonucleotide and the complementary Iowa Black FQ quencher modified oligonucleotide (Supplementary Data 1) at 95° C separately for 3 min and slow cooling (–0.1 °C s^−1^) to room temperature in annealing buffer (100 mM potassium acetate and 30 mM HEPES, pH 8.0). Annealed oligonucleotides where then purified by resolving them on 20% native PAGE–Tris-Borate-EDTA (TBE) gels, isolating the band of expected size and eluting at 4 °C overnight in annealing buffer. The eluted DNA gate was then ethanol-precipitated, resuspended in MilliQ ultrapure H_2_O and concentration quantified using the Thermo Scientific NanoDrop One Microvolume UV–Vis Spectrophotometer. The DNA gates used in Figs. 4–6 and Extended Data Fig. 6 and 7 were prepared using the same method but by annealing two complementary oligonucleotides without any modifications.

### Plasmids and genetic parts assembly

DNA oligonucleotides for cloning and sequencing were synthesized by Integrated DNA Technologies. Genes encoding aTFs were synthesized either as gBlocks (Integrated DNA Technologies) or gene fragments (Twist Bioscience). Protein expression plasmids were cloned using Gibson Assembly (NEB Gibson Assembly Master Mix, New England Biolabs, catalog no. E2611) into a pET-28c plasmid backbone and were designed to overexpress recombinant proteins as C terminus His-tagged fusions. A construct for expressing SmtB additionally incorporated a recognition sequence for cleavage and removal of the His-tag using TEV protease. Gibson assembled constructs were transformed into NEB Turbo cells, and isolated colonies were purified for plasmid DNA (QIAprep Spin Miniprep Kit, Qiagen, catalog no. 27106). Plasmid sequences were verified with Sanger DNA sequencing (Quintara Biosciences) using the primers listed in Supplementary Data 1.

All transcription templates were generated using one of the two methods: (1) PCR amplification (Phusion High-Fidelity PCR Kit, New England Biolabs, catalog no. E0553) of an oligo that includes a T7 promoter, an optional aTF operator site, the InvadeR coding sequence and an optional T7 terminator using the primer sets; or (2) annealing of two complementary oligonucleotides that include a T7 promoter, an optional aTF operator site, the InvadeR or RNA NOT gate coding sequences. All oligos and primer sets used in this study are listed in listed in Supplementary Data 1. Here, we define the T7 promoter as a minimal 17-bp sequence (TAATACGACTCACTATA) excluding the first G that is transcribed. The PCR-amplified templates were purified (QIAquick PCR purification kit, Qiagen, catalog no. 28106) and verified for the presence of a single DNA band of expected size on a 2% Tris-Acetate-EDTA–agarose gel. The templates generated by annealing two complementary oligos were prepared and purified using the same method described in ‘DNA gate preparation’. Concentrations of all DNA templates were determined using the Qubit dsDNA BR Assay Kit (Invitrogen, catalog no. Q32853).

All plasmids and DNA templates were stored at 4 °C until use. A spreadsheet listing the sequences and the Addgene accession numbers of all plasmids and oligos generated in this study are listed in Supplementary Data 1.

### RNA expression and purification

InvadeR variants used for the purified oligo binding assays were first expressed by an overnight IVT at 37 °C from a transcription template encoding a *cis*-cleaving hepatitis D ribozyme on the 3′-end of the InvadeR sequence with the following components: IVT buffer (40 mM Tris–HCl pH 8, 8 mM MgCl_2_, 10 mM dithiothreitol, 20 mM NaCl and 2 mM spermidine), 11.4 mM NTPs pH 7.5, 0.3 units (U) of thermostable inorganic pyrophosphatase (New England Biolabs, catalog no. M0296S), 100 nM transcription template, 50 ng of T7 RNAP and MilliQ ultrapure H_2_O to a total volume of 500 µl. The overnight IVT reactions were then ethanol-precipitated and purified by resolving them on a 20% urea–PAGE–TBE gel, isolating the band of expected size (26–29 nucleotides) and eluting at 4 °C overnight in MilliQ ultrapure H_2_O. The eluted InvadeR variants were ethanol-precipitated, resuspended in MilliQ ultrapure H_2_O, quantified using the Qubit RNA BR Assay Kit (Invitrogen, catalog no. Q10211) and stored at –20 °C until use. The hepatitis D ribozyme sequence used can be found in Supplementary Data 1.

### aTF expression and purification

aTFs were expressed and purified as previously described^7^. Briefly, sequence-verified pET-28c plasmids were transformed into the Rosetta 2(DE3)pLysS *E. coli* strain. Cell cultures (1–2 L) were grown in Luria broth at 37 °C, induced with 0.5 mM of isopropyl-β-d-thiogalactoside at an optical density (600 nm) of ~0.5 and grown for a further 4 h at 37 °C. Cultures were then pelleted by centrifugation and were either stored at –80 °C or resuspended in lysis buffer (10 mM Tris–HCl pH 8, 500 mM NaCl, 1 mM Tris(2-carboxyethyl)phosphine (TCEP) and protease inhibitor (Complete EDTA-free Protease Inhibitor Cocktail, Roche)) for purification. Resuspended cells were then lysed on ice through ultrasonication, and insoluble materials were removed by centrifugation. Clarified supernatant containing TetR was then purified using His-tag affinity chromatography with a Ni-NTA column (HisTrap FF 5 ml column, GE Healthcare Life Sciences) followed by size-exclusion chromatography (Superdex HiLoad 26/600 200 pg column, GE Healthcare Life Sciences) using an AKTAxpress fast protein liquid chromatography system. Clarified supernatants containing TtgR and SmtB were purified using His-tag affinity chromatography with a gravity column charged with Ni-NTA Agarose (Qiagen, catalog no. 30210). The eluted fractions from the fast protein liquid chromatography (for TetR) or from the gravity column (for TtgR and SmtB) were concentrated and buffer exchanged (25 mM Tris–HCl, 100 mM NaCl, 1 mM TCEP, 50% glycerol v/v) using centrifugal filtration (Amicon Ultra-0.5, Millipore Sigma). Protein concentrations were determined using the Qubit Protein Assay Kit (Invitrogen, catalog no. Q33212). The purity and size of the proteins were validated on an SDS–PAGE gel (Mini-PROTEAN TGX and Mini-TETRA cell, Bio-Rad). Purified proteins were stored at –20 °C.

### IVT reactions

Homemade IVT reactions were set up by adding the following components listed at their final concentration: IVT buffer (40 mM Tris–HCl pH 8, 8 mM MgCl_2_, 10 mM dithiothreitol, 20 mM NaCl and 2 mM spermidine), 11.4 mM NTPs pH 7.5, 0.3 U of thermostable inorganic pyrophosphatase (New England Biolabs, catalog no. M0296S), transcription template, DNA gate(s) and MilliQ ultrapure H_2_O to a total volume of 20 µl. Regulated IVT reactions additionally included a purified aTF at the indicated concentration and were equilibrated at 37 °C for ~10 min. Immediately before plate reader measurements, 2 ng of T7 RNAP and, optionally, a ligand at the indicated concentration were added to the reaction. Reactions were then characterized on a plate reader as described in ‘Plate reader quantification and micromolar equivalent fluorescein standardization’.

### RNA extraction from IVT reactions

For RNA products shown on the gel images of Extended Data Figs. 1c,f and 2c, IVT reactions were first set up as described above. Then, phenol–chloroform extraction followed by ethanol precipitation was performed to remove any proteins. The reactions were then rehydrated in 1× TURBO DNase buffer with 2 U of TURBO DNase (Invitrogen, catalog no. QAM2238) to a total volume of 20 µl and incubated at 37° C for 30 min to remove the DNA gates and the transcription templates. Phenol–chloroform extraction followed by ethanol precipitation was performed again to remove DNase and rehydrated in MilliQ ultrapure H_2_O. The concentrations of the extracted RNA products were measured using the Qubit RNA HS assay kit (Invitrogen, catalog no. Q32852) and stored in –20 °C until further analysis such as PAGE. For PAGE analysis of these extracted RNA products, 20% urea–PAGE–TBE gels were used, and the gels were imaged using a ChemiDoc Touch Gel Imaging System (Bio-Rad Image Lab Touch software v.1.2.0.12).

### Freeze-drying

Before lyophilization, PCR tube caps were punctured with a pin to create three holes. Lyophilization of ROSALIND reactions was then performed by assembling the components of IVT (above) with the addition of 50 mM sucrose and 250 mM d-mannitol. Assembled reaction tubes were immediately transferred into a prechilled aluminum block and placed in a –80 °C freezer for 10 min to allow slow-freezing. Following the slow-freezing, reaction tubes were wrapped in Kimwipes and aluminum foil, submerged in liquid nitrogen and then transferred to a FreeZone 2.5 L Bench Top Freeze Dry System (Labconco) for overnight freeze-drying with a condenser temperature of –85 °C and 0.04 mbar pressure. Unless rehydrated immediately, freeze-dried reactions were packaged as follows. The reactions were placed in a vacuum-sealable bag with a desiccant (Dri-Card Desiccants, Uline, catalog no. S-19582), purged with argon using an argon canister (ArT Wine Preserver, Amazon, catalog no. 8541977939) and immediately vacuum-sealed (KOIOS Vacuum Sealer Machine, Amazon, catalog no. TVS-2233). The vacuum-sealed bag then was placed in a light-protective bag (Mylar open-ended food bags, Uline, catalog no. S-11661), heat-sealed (Metronic 8-inch Impulse Bag Sealer, Amazon, catalog no. 8541949845) and stored in a cool, shaded area until use.

### Plate reader quantification and micromolar equivalent fluorescein standardization

A National Institute of Standards and Technology traceable standard (Invitrogen, catalog no. F36915) was used to convert arbitrary fluorescence measurements to micromolar equivalent fluorescein (MEF). Serial dilutions from a 50 µM stock were prepared in 100 mM sodium borate buffer at pH 9.5, including a 100 mM sodium borate buffer blank (total of 12 samples). For each concentration, nine replicates of samples were created in batches of three, and fluorescence values were read at an excitation wavelength of 495 nm and emission wavelength of 520 nm for 6-FAM (fluorescein)-activated fluorescence, or at an excitation wavelength of 472 nm and emission wavelength of 507 nm for 3-way junction dimeric broccoli (3WJdB)-activated fluorescence on a plate reader (Synergy H1, BioTek Gen5 v.2.04). Fluorescence values for a fluorescein concentration in which a single replicate saturated the plate reader were excluded from the analysis. The remaining replicates (nine per sample) were then averaged at each fluorescein concentration, and the average fluorescence value of the blank was subtracted from all values. Linear regression was then performed for concentrations within the linear range of fluorescence (0–3.125 µM fluorescein) between the measured fluorescence values in arbitrary units and the concentration of fluorescein to identify the conversion factor. For each plate reader, excitation, emission and gain setting, we found a linear conversion factor that was used to correlate arbitrary fluorescence values to MEF (Supplementary Fig. 1 and Supplementary Data 3).

For characterization, 19 µl of reactions were loaded onto a 384-well optically clear, flat-bottom plate using a multichannel pipette, covered with a plate seal and measured on a plate reader (Synergy H1, BioTek Gen5 v.2.04). Kinetic analysis of 6-FAM (fluorescein)-activated fluorescence was performed by reading the plate at 1-min intervals with excitation and emission wavelengths of 495 and 520 nm, respectively, for 2 h at 37 °C. Kinetic analysis of 3WJdB-activated fluorescence was performed by reading the plate at 3-min intervals with excitation and emission wavelengths of 472 and 507 nm, respectively, for 4 h at 37 °C. Arbitrary fluorescence values were then converted to MEF by dividing with the appropriate calibration conversion factor.

Except for the data in Fig. 6b, no background subtraction was performed when analyzing outputs from any reaction. An example of this standardization procedure is shown in Supplementary Fig. 1.

### Fluorescence data normalization (Fig. 6b only)

Data shown in Fig. 6b were generated as above and then normalized using the following method to compare experimental observations with ODE simulations. Raw fluorescence values were first standardized to MEF (µM fluorescein) using the method described above. The maximum MEF value was then determined among all of the reactions run (5 conditions × 3 replicates = 15 reactions). Each MEF value at every time interval was then normalized using the following formula:

$$f\left( x \right) = \frac{{{\mathrm{MEF}}_{t = x} - {\mathrm{MEF}}_{t = 0}}}{{{\mathrm{Max}}\,{\mathrm{MEF}} - {\mathrm{MEF}}_{t = 0}}}$$

$${\mathrm{where}}\,x\,{\mathrm{is}}\,{\mathrm{a}}\,{\mathrm{given}}\,{\mathrm{time}}\,{\mathrm{point}}\,\left( {0 \le x \le 120} \right)$$

Background subtraction was performed to account for the non-zero fluorescence observed for the quenched DNA signal gate. Once all data were normalized according to the formula above, *n* = 3 replicates per condition were averaged, and the corresponding standard deviation value per condition was calculated.

### Gel image analysis

Uncropped, unprocessed gel images presented in Supplementary Fig. 2d and Extended Data Figs. 1c,f and 2c are available either as source data or as Supplementary Data 2 and deposited in *Mendeley Data* (10.17632/hr3j3yztxb.1). The band intensity from a SYBR gold-stained urea–PAGE gel in Extended Data Fig. 2c was calculated with Fiji–ImageJ using the traditional lane-profile method as previously described^56^. Briefly, a region of interest in every lane was registered using a rectangle of the same dimensions. The uneven background was then accounted for by drawing a straight line at the bottom of each peak, and the peak area in each lane was calculated using the wand tool. The peak areas of the RNA standard were then plotted against the total amounts loaded to create the standard curve in Extended Data Fig. 2d (linear range: 0.25–2 ng). Using the conversion factor from the standard curve, the concentrations of InvadeR variants were estimated from the peak area values obtained from the wand tool.

### Tap and lake water sampling

For ZnSO_4_-spiked tap water from Evanston, IL, two bottles containing approximately 50 ml of the water samples were collected from a drinking fountain. One of the bottles was then filtered at 0.22 µm using a Steriflip-GP sterile vacuum filtration system (Millipore Sigma, catalog no. SCGP00525). Both the filtered and unfiltered water samples were spiked using 10, 1 or 0.1 mM ZnSO_4_ solution that has been diluted from the 2 M ZnSO_4_ solution stock (Sigma, catalog no. 83265). Upon rehydration, fluorescence measurements of the reactions were performed using a plate reader (see ‘Plate reader quantification and MEF standardization’). For ZnSO_4_-spiked Lake Michigan water from Evanston, IL, the same sampling method was applied.

### ODE simulations

ODEs for each reactant species were derived using aTF-binding, IVT and TMSD reaction kinetics. ODEs were calculated using an ODE solver function, *odeint* from the Scipy.Integrate package in Python v.3.7.6. Kinetic parameters were estimated from literature, and initial conditions were set to experimental conditions, with intermediates species set at zero. Details of the ODE simulations are discussed in Supplementary Information.

### Statistics and reproducibility

The number of replicates and types of replicates performed are described in the legend to each figure. Individual data points are shown, and where relevant, the average ± s.d. is shown; this information is provided in each figure legend. The type of statistical analysis performed in Fig. 3d and Extended Data Fig. 3 is described in the legend to each figure. Exact *P* values along with degrees of freedom computed from the statistical analysis can be found in the source data.

### Reporting Summary

Further information on research design is available in the Nature Research Reporting Summary linked to this article.

## Online content

Any methods, additional references, Nature Research reporting summaries, source data, extended data, supplementary information, acknowledgements, peer review information; details of author contributions and competing interests; and statements of data and code availability are available at 10.1038/s41589-021-00962-9.

## Supplementary information

Supplementary Information Supplementary Figs. 1–5 and Method on the ODE modeling used in this study.

Reporting Summary

Supplementary Data 1 DNA and protein sequences used in this study.

Supplementary Data 2 Raw urea–PAGE gel image shown in **Supplementary Fig. 2d**.

Supplementary Data 3 Calibrated plate reader data for supplementary figures.

Supplementary Data 4 Designs and sequences of all logic gates built in this study and their sources.

Supplementary Data 5 Experimental conditions (ligands, aTFs, DNA templates and DNA gates concentrations) used for all logic gates and their kinetic parameters used for the ODE simulations.

Supplementary Data 6 Jupyter notebook codes used to simulate results shown in Extended Data 5, Extended Data 8 and Fig. 6.

## Data Availability

All data presented in this paper are available as source data and as supplementary data. All source data as well as Supplementary Data 2 and 3 are also deposited in Mendeley Data (doi: 10.17632/hr3j3yztxb.1)^57^. All plasmids used in this paper are available in Addgene with the identifiers 140371, 140374, 140391 and 140395. Source data are provided with this paper.

## Source data

Source Data Fig. 2 Calibrated plate reader source data for Fig. 2.
Source Data Fig. 3 Calibrated plate reader source data for Fig. 3.
Source Data Fig. 4 Calibrated plate reader source data for Fig. 4.
Source Data Fig. 5 Calibrated plate reader source data for Fig. 5.
Source Data Fig. 6 Calibrated plate reader source data for Fig. 6.
Source Data Extended Data Fig. 1 Calibrated plate reader source data for Extended Data Fig. 1.
Source Data Extended Data Fig. 1 Unprocessed, uncropped urea–PAGE gels shown in Extended Data Fig. 1 and their short descriptions.
Source Data Extended Data Fig. 2 Calibrated plate reader source data for Extended Data Fig. 2.
Source Data Extended Data Fig. 2 Unprocessed, uncropped urea–PAGE gel shown in Extended Data Fig. 2 and its short description.
Source Data Extended Data Fig. 3 Calibrated plate reader source data for Extended Data Fig. 3.
Source Data Extended Data Fig. 4 Calibrated plate reader source data for Extended Data Fig. 4.
Source Data Extended Data Fig. 5 Calibrated plate reader source data for Extended Data Fig. 5.
Source Data Extended Data Fig. 6 Calibrated plate reader source data for Extended Data Fig. 6.
Source Data Extended Data Fig. 7 Calibrated plate reader source data for Extended Data Fig. 7.
Source Data Extended Data Fig. 8 Calibrated plate reader source data for Extended Data Fig. 8.
Source Data Extended Data Fig. 9 Calibrated plate reader source data for Extended Data Fig. 9.
Source Data Extended Data Fig. 10 Calibrated plate reader source data for Extended Data Fig. 10.

## Code availability

The Jupyter Notebook files with the Python codes used in Extended Data Fig. 5, Extended Data Fig. 8 and Fig. 6 are provided as Supplementary Data 6, and the ODE model used in this paper is described in the Supplementary Information. The Python codes are also available in GitHub at https://git.io/Jtlh1 (ref. ^58^).
