# Supplementary material for: Programming cell-free biosensors with DNA strand displacement circuits
Source: Nat Chem Biol. 2022 Feb 17;18(4):385–93. doi: 10.1038/s41589-021-00962-9 (PMC8964419; doi:10.1038/s41589-021-00962-9)
Supplement: Supplementary file 1 — Supplementary Figs. 1–5 and Method on the ODE modeling used in this study. [file 41589_2021_962_MOESM1_ESM.pdf]

nature portfolio

https://doi.org/10.1038/s41589-021-00962-9

Supplementary information

Programming cell-free biosensors with DNA strand displacement circuits

In the format provided by the authors and unedited

Supplementary Information for Programming Cell-Free Biosensors with DNA Strand Displacement Circuits

Jaeyoung K. Jung,[1,2,3] Chloé M. Archuleta,[1,2,3] Khalid K. Alam,[4] and Julius B. Lucks[1,2,3,4,5,%]

1 – Department of Chemical and Biological Engineering, Northwestern University (Evanston IL, USA)

2 – Center for Synthetic Biology, Northwestern University (Evanston, IL, USA)

3 – Center for Water Research, Northwestern University (Evanston, IL, USA)

4 – Stemloop, Inc. (Evanston, IL, USA)

5 – Interdisciplinary Biological Sciences Graduate Program, Northwestern University (Evanston, IL, USA)

% correspondence: jblucks@northwestern.edu

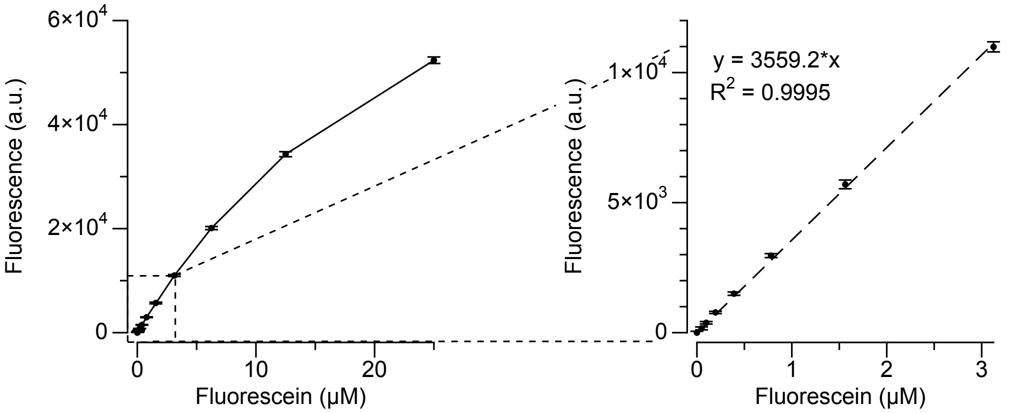

Supplementary Fig. 1 | Micromolar Equivalent Fluorescein (MEF) standardization. Arbitrary units of fluorescence were standardized to μM concentrations of fluorescein using a NIST traceable standard (see **Materials and Methods**). In the representative example shown here, a dilution series of fluorescein was prepared in buffer (100 mM sodium borate, pH 9.5) and measured on a plate reader using the same settings for measuring 6' FAM signal (495 nm excitation, 520 nm emission) from the DNA signal gates. The resulting curve, calculated over the linear range of 0–3.125 μM, was then used to standardize fluorescence measured from ROSALIND reactions with TMSD outputs. The standard curve was generated for each plate reader and each measurement setting (see **Materials and Methods**). Data are shown for n=9 replicates of each sample, which were prepared in three batches of three replicates. Error bars indicate the average of these replicates ± standard deviation (**Supplementary Data 3**).

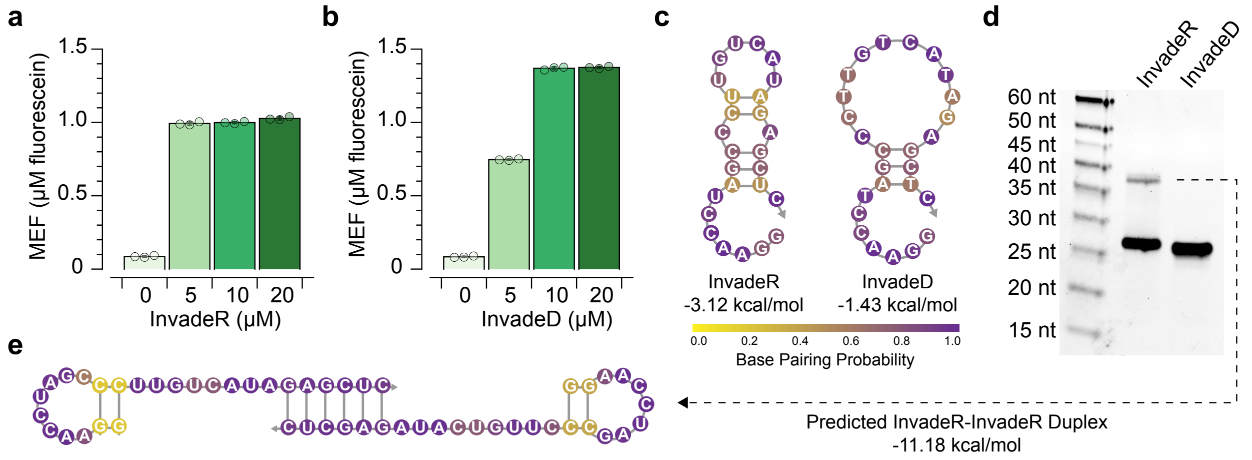

Supplementary Fig. 2 | TMSD by Invading RNA and DNA strands. Titration of a purified **a**, InvadeR (RNA) and **b**, InvadeD (DNA) into reactions containing 10 µM of the DNA signal gate in annealing buffer (100 mM potassium acetate, 30 mM HEPES) after 15 minutes. **c**, Secondary structures, minimum free energies and base pairing probabilities of the InvadeR and InvadeD molecules predicted by NUPACK[1] at 37° C. **d**, An urea-PAGE gel of purified InvadeR and InvadeD. The higher molecular weight band in the InvadeR lane likely corresponds to **e**, a duplex formed by two InvadeR molecules interacting with each other, as predicted by NUPACK[1]. Data shown in **a** and **b** are n=3 independent biological replicates each plotted as a point with raw fluorescence values standardized to MEF (µM fluorescein). Each bar height represents the average of the replicates, and error bars indicate the average of the replicates ± standard deviation. Data shown in **d** are a representative of n=3 independent biological replicates. The uncropped, unprocessed gel image shown in **d** is available as **Supplementary Data 2**.

1. Zadeh, J.N., et al., *NUPACK: Analysis and design of nucleic acid systems*. J Comput Chem, 2011. **32**(1): p. 170-3.

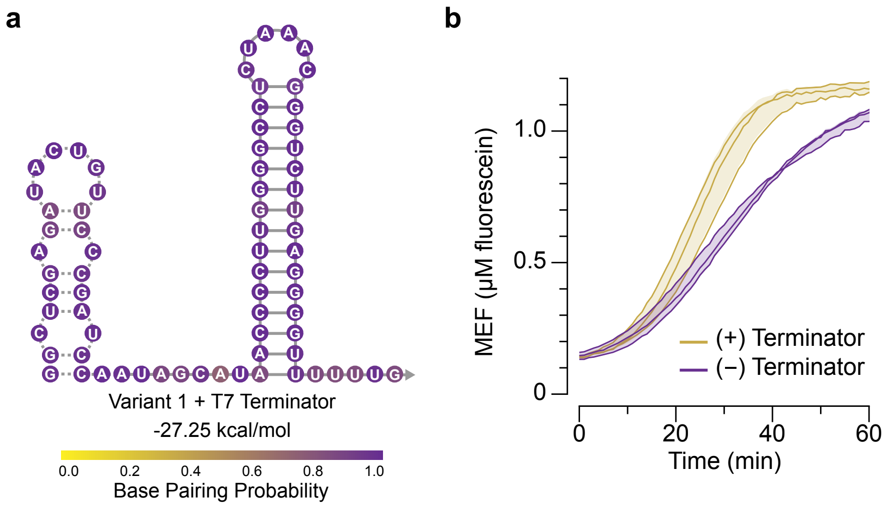

Supplementary Fig. 3 | Adding a T7 terminator does not notably improve the initial speed of ROSALIND with TMSD. a, Secondary structure, minimum free energy and base pairing probabilities of the InvadeR variant 1 (dashed backbone) that includes the T7 terminator (solid backbone) as predicted by NUPACK[1] at 37 °C. **b**, Comparison of the kinetics of InvadeR variant 1 with and without the T7 terminator. All data shown are n=3 independent biological replicates each plotted as a line with raw fluorescence values standardized to MEF (µM fluorescein). Shading indicates the average of the replicates ± standard deviation.

1. Zadeh, J.N., et al., *NUPACK: Analysis and design of nucleic acid systems*. J Comput Chem, 2011. **32**(1): p. 170-3.

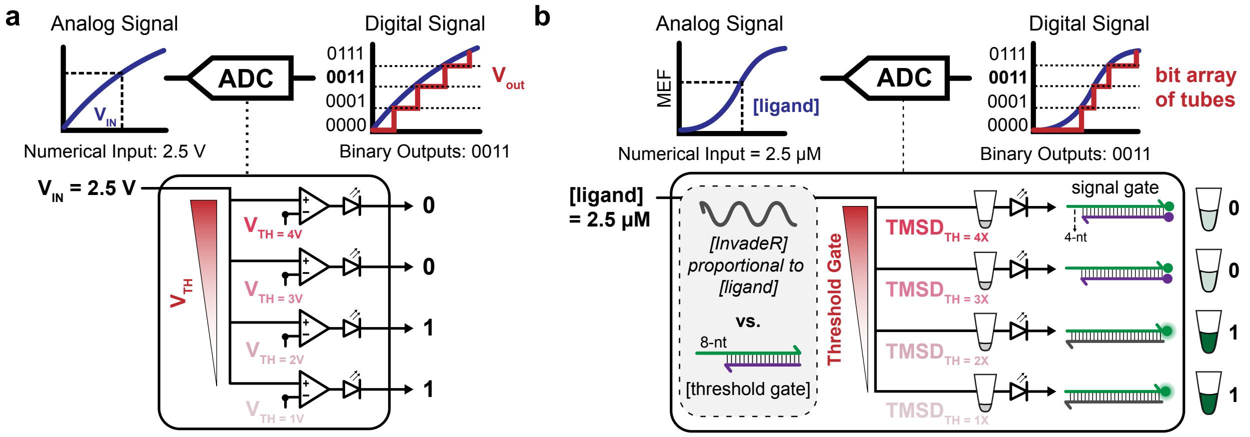

Supplementary Fig. 4 | A molecular analog-to-digital converter circuit enables ligand quantification. **a**, An electronic analog-to-digital converter (ADC) circuit takes in a numerical voltage value (analog input) and generates a binary output of 1s and 0s (digital output). It is built by configuring a series of comparator circuits, each comparing the input voltage to a variable reference voltage to produce a binary output of 1 if the input exceeds the reference value. By setting the threshold voltages (V_{TH}) of the comparators to be increasing along the series, the input voltage value is converted into an array of bits with more 1's representing a higher voltage. **b**, A molecular version of an ADC circuit can be built by implementing TMSD thresholding circuits that compare the input target ligand concentration to a pre-defined threshold value. By titrating the pre-defined threshold value, the molecular ADC circuit can generate different bit arrays to indicate the concentration range of the target ligand.

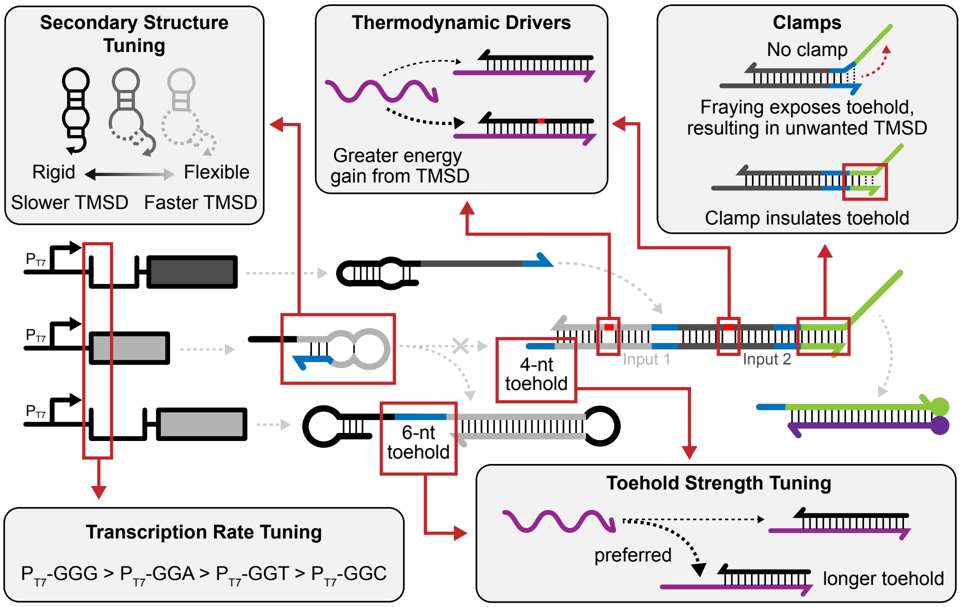

Supplementary Fig. 5 | Invading RNA Design Features. Using a NIMPLY gate as an example, we show five important design features of an Invading RNA strand for engineering RNA-based TMSD reactions. The transcription rate of the Invading RNA strand can be tuned by altering the initially transcribed sequences immediately following the T7 promoter sequence.[2] The secondary structure of the Invading RNA strand also plays an essential role in the kinetic reaction rate of TMSD – if the toehold binding region is sequestered within an RNA structure, it significantly slows down the strand displacement process. Invading RNAs can be preferentially biased to react with specific DNA gates over others by two different methods: 1) lengthening the toehold of the preferred gate to speed TMSD kinetics, and 2) introducing mismatches in the preferred gate to create a thermodynamic driver for sequestering the invading strand. Finally, clamps that insulate a toehold region from fraying are important in preventing leak from unwanted TMSD reactions.

2. Conrad, T., et al., *Maximizing transcription of nucleic acids with efficient T7 promoters*. Commun Biol, 2020. **3**(1): p. 439.

Supplementary Data File Descriptions

Jung_TMSD_ROSALIND_Supp_Data_File1_Sequences.xlsx – DNA and Protein sequences used in this study.

Jung_TMSD_ROSALIND_Supp_Data_File2_Gel_Image.zip – Raw Urea-PAGE gel image shown in **Supplementary Fig. 2d**.

Jung_TMSD_ROSALIND_Supp_Data_File3_SI_Source_Data.xlsx – Calibrated plate reader data for supplementary figures.

Jung_TMSD_ROSALIND_Supp_Data_File4_Logic_Gate_Designs.zip – Designs and sequences of all logic gates built in this study and their sources.

Jung_TMSD_ROSALIND_Supp_Data_File5_Logic_Gate_Conditions.xlsx – Experimental conditions (ligands, aTFs, DNA templates and DNA gates concentrations) used for all logic gates and their kinetic parameters used for the ODE simulations.

Jung_TMSD_ROSALIND_Supp_Data_File6_Jupyter_Notebooks.zip – Jupyter notebook codes used to simulate results shown in **Extended Data 5**, **Extended Data 8** and **Fig. 6**.

Supplementary Method for Programming Cell-Free Biosensors with DNA Strand Displacement Circuits

Jaeyoung K. Jung[1,2,3], Chloé M. Archuleta[1,2,3], Khalid K. Alam[4] and Julius B. Lucks[1,2,3,4,5,*]

1 – Department of Chemical and Biological Engineering, Northwestern University (Evanston IL, USA)
2 – Center for Synthetic Biology, Northwestern University (Evanston, IL, USA)
3 – Center for Water Research, Northwestern University (Evanston, IL, USA)
4 – Stemloop, Inc. (Evanston, IL, USA)
5 – Interdisciplinary Biological Sciences Graduate Program, Northwestern University (Evanston, IL, USA)
* - correspondence: jblucks@northwestern.edu

ODE Model of Basic Components of TMSD Logic Circuit

In this section, we model three representative logic gates that can be implemented with the ROSALIND-TMSD system using Ordinary Differential Equations. A similar workflow is used for each of the gates described in Extended Data 5.

ROSALIND-TMSD NOT Gate for Tetracycline

For the first system, we will model a NOT gate for tetracycline using a dual-template system. Equations are derived using kinetic rates of T7 RNAP-DNA binding, TetR-*tetO* binding, TetR-tetracycline binding, TMSD reactions, and T7 RNAP-mediated IVT reactions. The following variables will be used:

Abbreviation	Description
D_{unreg}	Unregulated DNA template
D_{tetO}	Regulated DNA template with tetO operator sequence
RNAP	T7 RNA Polymerase
RD_{unreg}	T7 RNAP and unregulated DNA template bound complex
RD_{tetO}	T7 RNAP and tetO-regulated DNA template bound complex
TetR	unbound, free TetR dimer
TetRD	TetR dimer bound to one tetO
tet	unbound, free tetracycline
TetR-tet	one tet ion bound to TetR dimer
$InvR_U$	transcribed invading RNA strand from unregulated template
$InvR_T$	transcribed invading RNA strand from tetO template
$Gated_T$	$InvR_T$ and $InvR_U$ bound complex
SD	fluorescent DNA strand from signal gate
Q	InvadeR and FAM heteroduplex
RQ	signal gate

In this model, we assume:

1. One-to-one binding of T7 RNAP and T7 promoter on the DNA template
2. The DNA template can be bound to either RNAP or TF, but not both.
3. One-to-one binding of TetR dimer and *tetO* on the DNA template
4. One-to-one binding of TetR dimer and a tetracycline ion [5]
5. TetR dimer can be bound to either *tetO* on the DNA template or tetracycline, but not both.
6. All TMSD reactions are irreversible.
7. Fraying within each gate is ignored.

With these assumptions, we have the following reactions and ODEs in the system:

Reactions:

Unregulated Template

$$RNAP + D_{unreg} \underset{k_{unbind}}{\overset{k_{bind}}{\longleftrightarrow}} RD_{unreg}$$

$$RD_{unreg} \xrightarrow{k_{m,unreg}} InvR_U + RNAP + D_{unreg}$$

tetO-Regulated Template

$$TetR + D_{tetO} \underset{k_{derep,T}}{\overset{k_{rep,T}}{\longleftrightarrow}} TetRD$$

$$TetR + tet \underset{k_{unin,T}}{\overset{k_{ind,T}}{\longleftrightarrow}} TetR\text{-}tet$$

$$RNAP + D_{tetO} \underset{k_{unbind}}{\overset{k_{bind}}{\longleftrightarrow}} RD_{tetO}$$

$$RD_{tetO} \xrightarrow{k_{m,tetO}} InvR_T + RNAP + D_{tetO}$$

Strand Displacements

$$InvR_T + InvR_U \xrightarrow{k_{SD,NOT}} Gated_T$$

$$InvR_U + RQ \xrightarrow{k_{SD,mis}} SD + Q$$

ODEs:

$$\frac{d[RNAP]}{dt} = k_{unbind}[RD_{tetO}] - k_{bind}[D_{tetO}][RNAP] + k_{m,tetO}[RD_{tetO}] + k_{unbind}[RD_{unreg}] - k_{bind}[D_{unreg}][RNAP] + k_{m,unreg}[RD_{unreg}]$$

$$\frac{d[D_{unreg}]}{dt} = k_{unbind}[RD_{unreg}] - k_{bind}[D_{unreg}][RNAP] + k_{m,unreg}[RD_{unreg}]$$

$$\frac{d[D_{tetO}]}{dt} = k_{unbind}[RD_{tetO}] - k_{bind}[D_{tetO}][RNAP] + k_{derep,T}[TetRD] - k_{rep,T}[TetR][D_{tetO}] + k_{m,tetO}[RD_{tetO}]$$

$$\frac{d[TetR]}{dt} = k_{derep,T}[TetRD] - k_{rep,T}[D_{tetO}][TetR] - k_{ind,T}[TetR][tet] + k_{unin,T}[TetR\text{-}tet]$$

$$\frac{d[TetRD]}{dt} = k_{rep,T}[D_{tetO}][TetR] - k_{derep,T}[TetRD]$$

$$\frac{d[tet]}{dt} = k_{unin,T}[TetR\text{-}tet] - k_{ind,T}[TetR][tet]$$

$$\frac{d[TetR\text{-}tet]}{dt} = k_{ind,T}[TetR][tet] - k_{unin,T}[TetR\text{-}tet]$$

$$\frac{d[RD_{tetO}]}{dt} = k_{bind}[D_{tetO}][RNAP] - k_{unbind}[RD_{tetO}] - k_{m,tetO}[RD_{tetO}]$$

$$\frac{d[RD_{unreg}]}{dt} = k_{bind}[D_{unreg}][RNAP] - k_{unbind}[RD_{unreg}] - k_{m,unreg}[RD_{unreg}]$$

$$\frac{d[InvR_T]}{dt} = k_{m,tetO}[RD_{tetO}] - k_{SD-NOT}[InvR_U][InvR_T]$$

$$\frac{d[InvR_U]}{dt} = k_{m,unreg}[RD_{unreg}] - k_{SD-NOT}[InvR_T][InvR_U] - k_{SD-mis}[InvR_U][RQ]$$

$$\frac{d[Gated_T]}{dt} = k_{SD-NOT}[InvR_T][InvR_U]$$

$$\frac{d[SD]}{dt} = k_{SD-mis}[RQ][InvR_U]$$

$$\frac{d[Q]}{dt} = d[SD]$$

$$\frac{d[RQ]}{dt} = -d[SD]$$

This set of ODEs was then run using an ODE solver function, *odeint* from the Scipy.Integrate package in Python 3.7.6. using the rate parameters shown below. The

initial concentrations were to set to experimental values also shown below, with all intermediates set to 0.

Parameters:

Parameter	Value	Reference and Note
$k_{m,tetO}$	0.1 /sec	[1]
$k_{m,unreg}$	0.03 /sec	see footnote **a**
k_{bind}	56 /µM-sec	[2]
k_{unbind}	0.2 /sec	[2]
k_{SD-mis}	0.004 /µM-sec	see footnote **b**
k_{SD-NOT}	0.08 /µM-sec	see footnote **c**
$k_{rep,T}$	2.98 /µM-sec	[4]
$k_{derep,T}$	0.001 /µM-sec	[4]
$k_{ind,T}$	4.0 /µM-sec	see footnote **d**
$k_{unin,T}$	0.022 /sec	see footnote **d**

Notes:

a This transcription rate is estimated to be 2.5 times slower than that of $k_{m,tetO}$ because the initially transcribed nucleotides have a slower transcriptional efficiency due to sequence [5].

b The estimated strand displacement rate for a 4-nt toehold is 0.04 /µM-sec [3]. To account for the energetic penalty from the encoded basepair mismatch between the unregulated transcript and the signal gate, we estimate a roughly 10-fold reduction in strand displacement rate as a result.

c The regulated RNA transcript encodes the matching base pair that the unregulated transcript lacks, so we estimate a roughly 2-fold increase in strand displacement rate as a result.

d These values were estimated to correlate with experimental results.

Initial Conditions:

Species	Value
$[D_{tetO}]$	0.1 µM
$[D_{unreg}]$	0.025 µM
$[RNAP]$	0.1 µM
$[TetR]$	5.0 µM
$[tet]$	20 µM
$[RQ]$	5.0 µM

References:

1. McClure, W. R., *Rate-limiting steps in RNA chain initiation.* PNAS, 1980. **77**(10): p.5634-8.
2. Ujvari, A. and C. T. Martin, *Thermodynamics and kinetic measurements of promoter biding by T7 RNA polymerase.* Biochemistry, 1996. **35**(46): p.14574-82.
3. Srinivas, N., et al., *On the biophysics and kinetics of toehold-mediated DNA strand displacement.* Nucleic Acids Research, 2013. **41**(22): p.10641-58
4. Kedracka-Krok, S. et al., *Kinetics and Equilibrium Studies of Tet Repressor-Operator Interaction.* Jounral of Protein Chemistry, 1998.
5. Conrad, T., et al., *Maximizing transcription of nucleic acids with efficient T7 promoters.* Commun. Biol., 2020. **3**(1):p. 439.

ROSALIND-TMSD AND Gate

For this system, we will model an AND gate for tetracycline and zinc using a dual-template system. Equations are derived using kinetic rates of T7 RNAP-DNA binding, TetR-*tetO* binding, TetR-tetracycline binding, SmtB-*smtO* binding, SmtB-Zn binding, TMSD reactions, and T7 RNAP-mediated IVT reactions. The following variables will be used:

Abbreviation	Description
D_{tetO}	Regulated DNA template with tetO operator sequence
D_{smtO}	Regulated DNA template with smtO operator sequence
RNAP	T7 RNA Polymerase
RD_{tetO}	T7 RNAP and tetO-regulated DNA template bound complex
TetR	unbound, free TetR dimer
RD_{smtO}	T7 RNAP and smtO-regulated DNA template bound complex
TetR	unbound, free TetR dimer
SmtB	unbound, free SmtB tetramer
TetRD	TetR dimer bound to one tetO
SmtBD	SmtB tetramer bound to one smtO
tet	unbound, free tetracycline
Zn	unbound, free zinc ion
TetR-tet	one tet ion bound to TetR dimer
SmtB-Zn	one Zn ion bound to SmtB tetramer
$InvR_T$	transcribed invading RNA strand from tetO template
$InvR_S$	transcribed invading RNA strand from smtO template
AND-Gate	dsDNA containing InvD and toeholds for $InvR_T$ and $InvR_S$
$InvR_D$	strand-displaced ssDNA from AND gate
$Gated_{TS}$	$InvR_T$ and $InvR_S$ bound to AND-Gate
SD	fluorescent DNA strand from signal gate
Q	InvadeR and FAM heteroduplex
RQ	signal gate

In this model, we assume:

1. One-to-one binding of T7 RNAP and T7 promoter on the DNA template
2. The DNA template can be bound to either RNAP or TF, but not both.
3. One-to-one binding of TetR dimer and *tetO* on the DNA template
4. One-to-one binding of SmtB tetramer and *smtO* on the DNA template (see footnote **a**)
5. One-to-one binding of TetR dimer and a tetracycline ion
6. One-to-one binding of SmtB tetramer and a Zn ion (see footnote **a**)

7. TetR dimer can be bound to either *tetO* on the DNA template or tetracycline, but not both.
8. SmtB tetramer can be bound to either *smtO* on the DNA template or Zn, but not both.
9. All TMSD reactions are irreversible.
10. InvD can only be strand displaced from AND gate with both $InvR_T$ and $InvR_S$
11. Fraying within each gate is ignored.

With these assumptions, we have the following reactions and ODEs in the system:

Reactions:

<u>tetO-regulated Template</u>

$$TetR + D_{tetO} \xleftrightarrow[k_{derep,T}]{k_{rep,T}} TetRD$$

$$TetR + tet \xleftrightarrow[k_{unin,T}]{k_{ind,T}} TetR\text{-}tet$$

$$RNAP + D_{tetO} \xleftrightarrow[k_{unbind}]{k_{bind}} RD_{tetO}$$

$$RD_{tetO} \xrightarrow{k_{m,tetO}} InvR_T + RNAP + D_{tetO}$$

<u>smtO-Regulated Template</u>

$$SmtB + D_{smtO} \xleftrightarrow[k_{derep,S}]{k_{rep,S}} SmtBD$$

$$SmtB + Zn \xrightarrow[k_{unin,S}]{k_{ind,S}} SmtB\text{-}Zn$$

$$RNAP + D_{smtO} \xrightarrow[k_{unbind}]{k_{bind}} RD_{smtO}$$

$$RD_{smtO} \xrightarrow{k_{m,smtO}} InvR_S + RNAP + D_{smtO}$$

<u>Strand Displacements</u>

$$InvR_T + InvR_S + AND\text{-}Gate \xrightarrow{k_{SD,AND}} Inv_D + Gated_{TS}$$

$$InvR_D + RQ \xrightarrow{k_{SD}} SD + Q$$

ODEs:

$$\frac{d[RNAP]}{dt} = k_{unbind}[RD_{tetO}] - k_{bind}[D_{tetO}][RNAP] + k_{m,tetO}[RD_{tetO}] + k_{unbind}[RD_{smtO}] - k_{bind}[D_{smtO}][RNAP] + k_{m,smtO}[RD_{smtO}]$$

$$\frac{d[D_{tetO}]}{dt} = k_{unbind}[RD_{tetO}] - k_{bind}[D_{tetO}][RNAP] + k_{derep_T}[TetRD] - k_{rep_T}[TetR][D_{tetO}] + k_{m,tetO}[RD_{tetO}]$$

$$\frac{d[D_{smtO}]}{dt} = k_{unbind}[RD_{smtO}] - k_{bind}[D_{smtO}][RNAP] + k_{derep_S}[SmtBD] - k_{rep_S}[SmtB][D_{smtO}] + k_{m,smtO}[RD_{smtO}]$$

$$\frac{d[TetR]}{dt} = k_{derep_T}[TetRD] - k_{rep_T}[D_{tetO}][TetR] - k_{ind_T}[TetR][tet] + k_{unin_T}[TetR\text{-}tet]$$

$$\frac{d[SmtB]}{dt} = k_{derep_S}[SmtBD] - k_{rep_S}[D_{smtO}][SmtB] - k_{ind_S}[SmtB][Zn] + k_{unin_S}[SmtB\text{-}Zn]$$

$$\frac{d[TetRD]}{dt} = k_{rep_T}[D_{tetO}][TetR] - k_{derep_T}[TetRD]$$

$$\frac{d[SmtBD]}{dt} = k_{rep_S}[D_{smtO}][SmtB] - k_{derep_S}[SmtBD]$$

$$\frac{d[tet]}{dt} = k_{unin_T}[TetR\text{-}tet] - k_{ind_T}[TetR][tet]$$

$$\frac{d[Zn]}{dt} = k_{unin_S}[SmtB\text{-}Zn] - k_{ind_S}[SmtB][Zn]$$

$$\frac{d[TetR\text{-}tet]}{dt} = k_{ind_T}[TetR][tet] - k_{unin_T}[TetR\text{-}tet]$$

$$\frac{d[SmtB\text{-}Zn]}{dt} = k_{ind_S}[SmtB][Zn] - k_{unin_S}[SmtB\text{-}Zn]$$

$$\frac{d[RD_{tetO}]}{dt} = k_{bind}[D_{tetO}][RNAP] - k_{unbind}[RD_{tetO}] - k_{m,tetO}[RD_{tetO}]$$

$$\frac{d[RD_{smtO}]}{=} k_{bind}[D_{smtO}][RNAP] - k_{unbind}[RD_{smtO}] - k_{m,smtO}[RD_{smtO}]$$

$$\frac{d[InvR_T]}{dt} = k_{m,tetO}[RD_{tetO}] - k_{SD-AND}[InvR_S][InvR_T][AND\text{-}Gate]$$

$$\frac{d[InvR_S]}{dt} = k_{m,smtO}[RD_{smtO}] - k_{SD-AND}[InvR_S][InvR_T][AND\text{-}Gate]$$

$$\frac{d[InvD]}{dt} = k_{SD-AND}[InvR_S][InvR_T][AND\text{-}Gate] - k_{SD}[InvD][RQ]$$

$$\frac{d[AND\text{-}Gate]}{dt} = -k_{SD-AND}[InvR_S][InvR_T][AND\text{-}Gate]$$

$$\frac{d[SD]}{dt} = k_{SD}[InvD][RQ]$$

$$\frac{d[Q]}{dt} = d[SD]$$

$$\frac{d[RQ]}{dt} = -d[SD]$$

This set of ODEs was then run using an ODE solver function, *odeint* from the Scipy.Integrate package in Python 3.7.6. using the rate parameters shown below. The initial concentrations were to set to experimental values also shown below, with all intermediates set to 0.

Parameters:

Parameter	Value	Reference and Note
$k_{m,tetO}$	0.1 /sec	[1]
$k_{m,smtO}$	0.04 /sec	see footnote **b**
k_{bind}	56 /µM-sec	[2]
k_{unbind}	0.2 /sec	[2]
k_{SD}	0.04 /µM-sec	[3]
k_{SD-AND}	0.08 /µM-sec	see footnote **c**
$k_{rep,T}$	2.98 /µM-sec	[4]
$k_{derep,T}$	0.001 /µM-sec	[4]
$k_{ind,T}$	4.0 /µM-sec	see footnote **d**
$k_{unin,T}$	0.022 /sec	see footnote **d**
$k_{rep,S}$	3.0 /µM-sec	see footnote **e**
$k_{derep,S}$	0.18 /µM-sec	see footnote **e**
$k_{ind,S}$	80 /µM-sec	see footnote **f**
$k_{unin,S}$	0.1 /sec	see footnote **f**

Notes:

a We have found conflicting evidence from literature that reports different DNA-SmtB-zinc binding mechanisms. For instance, *Kar, S. R., et al* reports that SmtB predominantly forms a dimer and binds two zinc ions per subunit (therefore, one SmtB dimer binding 4 total zinc ions) [5]. On the other hand, *VanZile, M. L. et al* reports that SmtB binds one zinc ion per monomer [6]. A more recent literature from *Busenlenher, L. S. et al* reports that two SmtB dimers tightly bind a single

12-2-12 inverted repeat of the *smtO* sequence [7]. We have found that following the mechanism from the most recent literature [7] where SmtB forms a tetramer to bind a single *smtO* site matches our experimental observations the best. We also suspect that while a single SmtB tetramer can bind multiple zinc ions at a time, not all zinc ions are needed to induce the transcription regulated by the SmtB tetramer. Following this logic, we used a model where one zinc ion can bind to the SmtB tetramer to induce the transcription of the invading RNA, which precisely matched our experimental observations.

b This transcription rate is estimated to be 2 times slower than that of $k_{m,tetO}$ because the initially transcribed nucleotides have a slower transcriptional efficiency due to sequence [8].

c The estimated strand displacement rate for a 4-nt toehold is 0.04 /µM-sec [3]. To account for the favorable energetics from the increased basepairs of the AND gate, we estimate a roughly 2-fold increase in strand displacement rate as a result.

d These values were estimated to correlate with experimental results.

e There was no literature with this information available to the best of our knowledge. We have decided to estimate these values based on a reasonable range of a K_d value for a transcriptional repressor, which is typically in the nanomolar range. Currently, the rate constants are set so that the $K_d = \frac{k_{derepress}}{k_{repress}} \approx 60nM$.

f These values have been estimated to fit the association constants of SmtB-Zinc reported in [9].

Initial Conditions:

Species	Value
[D_{tetO}]	0.05 µM
[D_{smt}]	0.05 µM
[RNAP]	0.1 µM
[TetR]	3.75 µM
[SmtB]	3.75 µM
[tet]	30. µM
[Zn]	20. uM
[AND-Gate]	5.0 µM
[RQ]	5.0 µM

References:

1. McClure, W. R., *Rate-limiting steps in RNA chain initiation.* PNAS, 1980. **77**(10): p.5634-8.
2. Ujvari, A. and C. T. Martin, *Thermodynamics and kinetic measurements of promoter biding by T7 RNA polymerase.* Biochemistry, 1996. **35**(46): p.14574-82.
3. Srinivas, N., et al., *On the biophysics and kinetics of toehold-mediated DNA strand displacement.* Nucleic Acids Research, 2013. **41**(22): p.10641-58
4. Kedracka-Krok, S. et al., *Kinetics and Equilibrium Studies of Tet Repressor-Operator Interaction.* Jounral of Protein Chemistry, 1998.
5. Kar, S. R., et al., *The cyanobacterial repressor SmtB is predominantly a dimer and binds two Zn2+ ions per subunit.* Biochemistry, 1997. **36**(49): p.15343–8.
6. VanZile, M. L., et al., *The Zinc Metalloregulatory Protein Synechococcus PCC7942 SmtB Binds a Single Zinc Ion per Monomer with High Affinity in a Tetrahedral Coordination Geometry.* Biochemistry, 2000. **39**(38): p.11818-29.
7. Busenlehner, L. S., et al., *The SmtB/ArsR family of metalloregulatory transcriptional repressors: structural insights into prokaryotic metal resistance.* FEMS Microbiology Reviews, 2003. **27**: p.131-143.
8. Conrad, T., et al., *Maximizing transcription of nucleic acids with efficient T7 promoters.* Commun. Biol., 2020. **3**(1):p. 439.
9. VanZile, M. L., *Structural characterization of distinct a3N and a5 metal sites in the cyanobacterial zinc sensor SmtB.* Biochemistry (2002) **41**(31): p.9765-75

ROSALIND-TMSD OR Gate

For this system, we will model a OR gate for tetracycline and zinc using a dual-template system. Equations are derived using kinetic rates of T7 RNAP-DNA binding, TetR-*tetO* binding, TetR-tetracycline binding, SmtB-*smtO* binding, SmtB-Zn binding, TMSD reactions, and T7 RNAP-mediated IVT reactions. The following variables will be used:

Abbreviation	Description
D_{tetO}	Regulated DNA template with tetO operator sequence
D_{smtO}	Regulated DNA template with smtO operator sequence
RNAP	T7 RNA Polymerase
RD_{tetO}	T7 RNAP and tetO-regulated DNA template bound complex
TetR	unbound, free TetR dimer
RD_{smtO}	T7 RNAP and smtO-regulated DNA template bound complex
TetR	unbound, free TetR dimer
SmtB	unbound, free SmtB tetramer
TetRD	TetR dimer bound to one tetO
SmtBD	SmtB tetramer bound to one smtO
tet	unbound, free tetracycline
Zn	unbound, free zinc ion
TetR-tet	one tet ion bound to TetR dimer
SmtB-Zn	one zn ion bound to SmtB tetramer
$InvR_T$	transcribed invading RNA strand from tetO template
$InvR_S$	transcribed invading RNA strand from smtO template
OR_T	dsDNA with toehold for $InvR_T$
OR_S	dsDNA with toehold for $InvR_S$
$InvR_D$	strand-displaced ssDNA from OR gates
$Gated_T$	$InvR_T$ bound to OR_T
$Gated_S$	$InvR_S$ bound to OR_S
SD	fluorescent DNA strand from signal gate
Q	InvadeR and FAM heteroduplex
RQ	signal gate

In this model, we assume:

1. One-to-one binding of T7 RNAP and T7 promoter on the DNA template
2. The DNA template can be bound to either RNAP or TF, but not both.
3. One-to-one binding of TetR dimer and *tetO* on the DNA template
4. One-to-one binding of SmtB tetramer and *smtO* on the DNA template (see footnote **a**)
5. One-to-one binding of TetR dimer and a tetracycline ion

6. One-to-one binding of SmtB tetramer and a Zn ion (see footnote **a**)
7. TetR dimer can be bound to either *tetO* on the DNA template or tetracycline, but not both.
8. SmtB tetramer can be bound to either *smtO* on the DNA template or Zn, but not both.
9. All TMSD reactions are irreversible.
10. Fraying within each gate is ignored.

With these assumptions, we have the following reactions and ODEs in the system:

Reactions:

<u>tetO-regulated Template</u>

$$TetR + D_{tetO} \quad \underset{k_{derep,T}}{\overset{k_{rep,T}}{\longleftrightarrow}} \quad TetRD$$

$$TetR + tet \quad \underset{k_{unin,T}}{\overset{k_{ind,T}}{\longleftrightarrow}} \quad TetR\text{-}tet$$

$$RNAP + D_{tetO} \quad \underset{k_{unbind}}{\overset{k_{bind}}{\longleftrightarrow}} \quad RD_{tetO}$$

$$RD_{tetO} \quad \xrightarrow{k_{m,tetO}} \quad InvR_T + RNAP + D_{tetO}$$

<u>smtO-Regulated Template</u>

$$SmtB + D_{smtO} \quad \underset{k_{derep,S}}{\overset{k_{rep,S}}{\longleftrightarrow}} \quad SmtBD$$

$$SmtB + Zn \quad \underset{k_{unin,S}}{\overset{k_{ind,S}}{\longrightarrow}} \quad SmtB\text{-}Zn$$

$$RNAP + D_{smtO} \quad \underset{k_{unbind}}{\overset{k_{bind}}{\longrightarrow}} \quad RD_{smtO}$$

$$RD_{smtO} \quad \xrightarrow{k_{m,smtO}} \quad InvR_S + RNAP + D_{smtO}$$

<u>Strand Displacements</u>

$$InvR_T + OR_T \quad \xrightarrow{k_{SD,T}} \quad Inv_D + Gated_T$$

$$InvR_S + OR_S \quad \xrightarrow{k_{SD,S}} \quad Inv_D + Gated_S$$

$$InvR_D + RQ \quad \xrightarrow{k_{SD}} \quad SD + Q$$

ODEs:

$$\frac{d[RNAP]}{dt} = k_{unbind}[RD_{tetO}] - k_{bind}[D_{tetO}][RNAP] + k_{m,tetO}[RD_{tetO}] + k_{unbind}[RD_{smtO}] - k_{bind}[D_{smtO}][RNAP] + k_{m,smtO}[RD_{smtO}]$$

$$\frac{d[D_{tetO}]}{dt} = k_{unbind}[RD_{tetO}]-k_{bind}[D_{tetO}][RNAP]+k_{derep_T}[TetRD]-k_{rep_T}[TetR][D_{tetO}]+k_{m,tetO}[RD_{tetO}]$$

$$\frac{d[D_{smtO}]}{dt} = k_{unbind}[RD_{smtO}]-k_{bind}[D_{smtO}][RNAP]+k_{derep_S}[SmtBD]-k_{rep_S}[SmtB][D_{smtO}]+k_{m,smtO}[RD_{smtO}]$$

$$\frac{d[TetR]}{dt} = k_{derep_T}[TetRD] - k_{rep_T}[D_{tetO}][TetR] - k_{ind_T}[TetR][tet] + k_{unin_T}[TetR\text{-}tet]$$

$$\frac{d[SmtB]}{dt} = k_{derep_S}[SmtBD] - k_{rep_S}[D_{smtO}][SmtB] - k_{ind_S}[SmtB][Zn] + k_{unin_S}[SmtB\text{-}Zn]$$

$$\frac{d[TetRD]}{dt} = k_{rep_T}[D_{tetO}][TetR] - k_{derep_T}[TetRD]$$

$$\frac{d[SmtBD]}{dt} = k_{rep_S}[D_{smtO}][SmtB] - k_{derep_S}[SmtBD]$$

$$\frac{d[tet]}{dt} = k_{unin_T}[TetR\text{-}tet] - k_{ind_T}[TetR][tet]$$

$$\frac{d[Zn]}{dt} = k_{unin_S}[SmtB\text{-}Zn] - k_{ind_S}[SmtB][Zn]$$

$$\frac{d[TetR\text{-}tet]}{dt} = k_{ind_T}[TetR][tet] - k_{unin_T}[TetR\text{-}tet]$$

$$\frac{d[SmtB\text{-}Zn]}{dt} = k_{ind_S}[SmtB][Zn] - k_{unin_S}[SmtB\text{-}Zn]$$

$$\frac{d[RD_{tetO}]}{dt} = k_{bind}[D_{tetO}][RNAP] - k_{unbind}[RD_{tetO}] - k_{m,tetO}[RD_{tetO}]$$

$$\frac{d[RD_{smtO}]}{=} k_{bind}[D_{smtO}][RNAP] - k_{unbind}[RD_{smtO}] - k_{m,smtO}[RD_{smtO}]$$

$$\frac{d[InvR_S]}{dt} = k_{m,smtO}[RD_{smtO}] - k_{SD,S}[OR_S][InvR_S]$$

$$\frac{d[InvR_T]}{dt} = k_{m,tetO}[RD_{tetO}] - k_{SD,T}[OR_T][InvR_T]$$

$$\frac{d[OR_S]}{dt} = -k_{SD,S}[InvR_S][OR_S]$$

$$\frac{d[OR_T]}{dt} = -k_{SD,T}[InvR_T][OR_T]$$

$$\frac{d[InvD]}{dt} = k_{SD,S}[OR_S][InvR_S] + k_{SD,T}[OR_T][InvR_T] - k_{SD}[InvD][RQ]$$

$$\frac{d[SD]}{dt} = k_{SD}[RQ][InvD]$$

$$\frac{d[Q]}{dt} = d[SD]$$

$$\frac{d[RQ]}{dt} = -d[SD]$$

$$\frac{d[Gated_S]}{dt} = k_{SD,S}[InvR_S][OR_S]$$

$$\frac{d[Gated_T]}{dt} = k_{SD,T}[InvR_T][OR_T]$$

This set of ODEs was then run using an ODE solver function, *odeint* from the Scipy.Integrate package in Python 3.7.6. using the rate parameters shown below. The initial concentrations were to set to experimental values also shown below, with all intermediates set to 0.

Parameters:

Parameter	Value	Reference and Note
$k_{m,tetO}$	0.1 /sec	[1]
$k_{m,smtO}$	0.05 /sec	see footnote **b**
k_{bind}	56 /µM-sec	[2]
k_{unbind}	0.2 /sec	[2]
k_{SD}	0.04 /µM-sec	[3]
$k_{SD,S}$	0.04 /µM-sec	[3]
$k_{SD,T}$	0.04 /µM-sec	[3]
$k_{rep,T}$	2.98 /µM-sec	[4]
$k_{derep,T}$	0.001 /µM-sec	[4]
$k_{ind,T}$	4.0 /µM-sec	see footnote **c**
$k_{unin,T}$	0.022 /sec	see footnote **c**
$k_{rep,S}$	3.0 /µM-sec	see footnote **d**
$k_{derep,S}$	0.18 /µM-sec	see footnote **d**
$k_{ind,S}$	80 /µM-sec	see footnote **e**
$k_{unin,S}$	0.1 /sec	see footnote **e**

Notes:

a We have found conflicting evidence from literature that reports different DNA-SmtB-zinc binding mechanisms. For instance, *Kar, S. R., et al* reports that SmtB predominantly forms a dimer and binds two zinc ions per subunit (therefore, one SmtB dimer binding 4 total zinc ions) [5]. On the other hand, *VanZile, M. L. et al* reports that SmtB binds one zinc ion per monomer [6]. A more recent literature from *Busenlenher, L. S. et al* reports that two SmtB dimers tightly bind a single 12-2-12 inverted repeat of the *smtO* sequence [7]. We have found that following the mechanism from the most recent literature [7] where SmtB forms a tetramer to bind a single *smtO* site matches our experimental observations the best. We also suspect that while a single SmtB tetramer can bind multiple zinc ions at a time, not all zinc ions are needed to induce the transcription regulated by the SmtB tetramer. Following this logic, we used a model where one zinc ion can bind to the SmtB tetramer to induce the transcription of the invading RNA, which precisely matched our experimental observations.

b This transcription rate is estimated to be 2 times slower than that of $k_{m,tetO}$ because the initially transcribed nucleotides have a slower transcriptional efficiency due to sequence [8].

c These values were estimated to correlate with experimental results.

d There was no literature with this information available to the best of our knowledge. We have decided to estimate these values based on a reasonable range of a K_d value for a transcriptional repressor, which is typically in the nanomolar range. Currently, the rate constants are set so that the $K_d = \frac{k_{derepress}}{k_{repress}} \approx 60nM$.

e These values have been estimated to fit the association constants of SmtB-Zinc reported in [9].

Initial Conditions:

Species	Value
$[\text{D}_{tetO}]$	0.05 µM
$[\text{D}_{smt}]$	0.05 µM
[RNAP]	0.1 µM
[TetR]	2.5 µM
[SmtB]	5.0 µM
[tet]	30. µM
[Zn]	20. µM
$[\text{OR}_S]$	10.0 µM
$[\text{OR}_T]$	10.0 µM
[RQ]	5.0 µM

References:

1. McClure, W. R., *Rate-limiting steps in RNA chain initiation.* PNAS, 1980. **77**(10): p.5634-8.
2. Ujvari, A. and C. T. Martin, *Thermodynamics and kinetic measurements of promoter biding by T7 RNA polymerase.* Biochemistry, 1996. **35**(46): p.14574-82.
3. Srinivas, N., et al., *On the biophysics and kinetics of toehold-mediated DNA strand displacement.* Nucleic Acids Research, 2013. **41**(22): p.10641-58
4. Kedracka-Krok, S. et al., *Kinetics and Equilibrium Studies of Tet Repressor-Operator Interaction.* Jounral of Protein Chemistry, 1998.
5. Kar, S. R., et al., *The cyanobacterial repressor SmtB is predominantly a dimer and binds two Zn2+ ions per subunit.* Biochemistry, 1997. **36**(49): p.15343–8.
6. VanZile, M. L., et al., *The Zinc Metalloregulatory Protein Synechococcus PCC7942 SmtB Binds a Single Zinc Ion per Monomer with High Affinity in a Tetrahedral Coordination Geometry.* Biochemistry, 2000. **39**(38): p.11818-29.
7. Busenlehner, L. S., et al., *The SmtB/ArsR family of metalloregulatory transcriptional repressors: structural insights into prokaryotic metal resistance.* FEMS Microbiology Reviews, 2003. **27**: p.131-143.
8. Conrad, T., et al., *Maximizing transcription of nucleic acids with efficient T7 promoters.* Commun. Biol., 2020. **3**(1):p. 439.
9. VanZile, M. L., *Structural characterization of distinct a3N and a5 metal sites in the cyanobacterial zinc sensor SmtB.* Biochemistry (2002) **41**(31): p.9765-75

ODE Model of TMSD Thresholding Circuit

Here, we use the kinetic rates of T7 RNAP-DNA binding, SmtB-*smtO* binding, SmtB-Zn binding, TMSD reactions and T7 RNAP-mediated IVT reactions to simulate ROSALIND Reactions. The following variables will be used:

Abbreviation	Description
D	DNA template
RNAP	T7 RNA Polymerase
RD	T7 RNAP and DNA template bound complex
m	InvadeR
TF	Unbound, free SmtB tetramer
TFD	SmtB tetramer bound to one *smtO*
I	Unbound, free zinc ions
TFI	One zinc ion bound to SmtB tetramer
RQ	Signal gate
SD	InvadeR and 6'FAM heteroduplex
Q	Quencher DNA strand
Th	Threshold gate
SDTh	InvadeR and threshold heteroduplex
QTh	Incumbent strand from threshold gate

In this model, we assume:

1. One-to-one binding of T7 RNAP and T7 promoter on the DNA template
2. The DNA template can be bound to either RNAP or TF, but not both.
3. One-to-one binding of SmtB tetramer and *smtO* on the DNA template (see footnote **a**)
4. One-to-one binding of SmtB tetramer and a zinc ion (see footnote **a**)
5. SmtB tetramer can be bound to either *smtO* on the DNA template or zinc, but not both.
6. All TMSD reactions are irreversible.
7. Fraying within each gate is ignored.

With these assumptions, we have the following reactions and ODEs in the system:

Reactions:

$$RNAP + D \underset{k_{unbind}}{\overset{k_{bind}}{\longleftrightarrow}} RD$$

$$TF + D \underset{k_{derepress}}{\overset{k_{repress}}{\longleftrightarrow}} TFD$$

$$TF + I \xrightleftharpoons[k_{uninduce}]{k_{induce}} TFI$$

$$RD \xrightarrow{k_m} m + RNAP + D$$

$$m + RQ \xrightarrow{k_{SD}} SD + Q$$

$$m + Th \xrightarrow{k_{SDTh}} SDTh + QTh$$

ODEs:

$$\frac{d[RNAP]}{dt} = k_{unbind}[RD] - k_{bind}[RNAP][D] + k_m[RD]$$

$$\frac{d[RD]}{dt} = k_{bind}[RNAP][D] - k_{unbind}[RD] - k_m[RD]$$

$$\frac{d[D]}{dt} = k_{unbind}[RD] - k_{bind}[RNAP][D] + k_{derepress}[TFD] - k_{repress}[TF][D] + k_m[RD]$$

$$\frac{d[m]}{dt} = k_m[RD] - k_{SD}[RQ][m] - k_{SDTh}[Th][m]$$

$$\frac{d[TF]}{dt} = k_{derepress}[TFD] - k_{repress}[TF][D] - k_{induce}[TF][I] + k_{uninduce}[TFI]$$

$$\frac{d[TFD]}{dt} = k_{repress}[TF][D] - k_{derepress}[TFD]$$

$$\frac{d[I]}{dt} = k_{uninduce}[TFI] - k_{induce}[TF][I]$$

$$\frac{d[TFI]}{dt} = k_{induce}[TF][I] - k_{uninduce}[TFI]$$

$$\frac{d[RQ]}{dt} = -k_{SD}[RQ][m]$$

$$\frac{d[SD]}{dt} = k_{SD}[RQ][m]$$

$$\frac{d[Q]}{dt} = k_{SD}[RQ][m]$$

$$\frac{d[Th]}{dt} = -k_{SDTh}[Th][m]$$

$$\frac{d[SDTh]}{dt} = k_{SDTh}[Th][m]$$

$$\frac{d[QTh]}{dt} = k_{SDTh}[Th][m]$$

This set of ODEs was then run using an ODE solver function, *odeint* from the Scipy.Integrate package in Python 3.7.6. using the rate parameters shown below. For the unregulated reactions shown in Fig. 6b, the initial concentrations of TF, TFD, TFI and I were set to zero.

Parameters:

Parameter	Value	Reference and Note
k_m for T7-GG-InvR2	0.1 /sec	[1] see footnote **b**
k_m for T7-GG-smtO-InvR2	0.03 /sec	[1] see footnote **b**
k_{bind}	56 /µM-sec	[2]
k_{unbind}	0.2 /sec	[2]
k_{SD}	0.04 /µM-sec	[3] for 4-nt toehold
k_{SDTh}	4.0 /µM-sec	[3] for 8-nt toehold
$k_{repress}$	3.0 /µM-sec	see footnote **c**
$k_{derepress}$	0.18 /sec	see footnote **c**
k_{induce}	80 /µM-sec	[4] see footnote **d**
$k_{uninduce}$	0.1 /sec	[4] see footnote **d**

Notes:

a We have found conflicting evidence from literature that reports different DNA-SmtB-zinc binding mechanisms. For instance, *Kar, S. R., et al* reports that SmtB predominantly forms a dimer and binds two zinc ions per subunit (therefore, one SmtB dimer binding 4 total zinc ions) [5]. On the other hand, *VanZile, M. L. et al* reports that SmtB binds one zinc ion per monomer [6]. A more recent literature from *Busenlenher, L. S. et al* reports that two SmtB dimers tightly bind a single 12-2-12 inverted repeat of the *smtO* sequence [7]. We have found that following the mechanism from the most recent literature [7] where SmtB forms a tetramer to bind a single *smtO* site matches our experimental observations the best. We also suspect that while a single SmtB tetramer can bind multiple zinc ions at a time, not all zinc ions are needed to induce the transcription regulated by the SmtB tetramer. Following this logic, we used a model where one zinc ion can bind to the SmtB tetramer to induce the transcription of the invading RNA, which precisely matched our experimental observations.

b These values were adjusted from the reported rate to accommodate for different initially transcribed nucleotides in each template. For instance, pT7-GGGA has a much greater transcription efficiency than pT7-GGCA [8].

c There was no literature with this information available to the best of our knowledge. We have decided to estimate these values based on a reasonable range of a K_d value for a transcriptional repressor, which is typically in the nanomolar range. Currently, the rate constants are set so that the $K_d = \frac{k_{derepress}}{k_{repress}} \approx 60nM$.

d These values have been estimated to fit the association constants of SmtB-Zinc reported in *VanZile, et al* [4].

Initial Conditions For Unregulated Reaction (Fig. 6b):

Species	Value
[D]	0.05 µM
[RNAP]	0.1 µM
[Th]	0, 5, 10, 20, 40 µM
[RQ]	5.0 µM

Initial Conditions For Regulated Reaction (Fig. 6d):

Species	Value
[D]	0.05 µM
[RNAP]	0.1 µM
[TF]	5.0 µM
[I]	2, 3.5, 5, 10 µM
[Th]	0, 2.5, 5.0, 7.5 µM
[RQ]	2.5 µM

References:

1. McClure, W. R., *Rate-limiting steps in RNA chain initiation.* PNAS, 1980. **77**(10): p.5634-8.
2. Ujvari, A. and C. T. Martin, *Thermodynamics and kinetic measurements of promoter biding by T7 RNA polymerase.* Biochemistry, 1996. **35**(46): p.14574-82.
3. Srinivas, N., et al., *On the biophysics and kinetics of toehold-mediated DNA strand displacement.* Nucleic Acids Research, 2013. **41**(22): p.10641-58
4. VanZile, M. L., et al., *Structural characterization of distinct a3N and a5 metal sites in the cyanobacterial zinc sensor SmtB.* Biochemistry, 2002. **41**(31): p.9765-75
5. Kar, S. R., et al., *The cyanobacterial repressor SmtB is predominantly a dimer and binds two Zn2+ ions per subunit.* Biochemistry, 1997. **36**(49): p.15343–8.

6. VanZile, M. L., et al., *The Zinc Metalloregulatory Protein Synechococcus PCC7942 SmtB Binds a Single Zinc Ion per Monomer with High Affinity in a Tetrahedral Coordination Geometry*. Biochemistry, 2000. **39**(38): p.11818-29.
7. Busenlehner, L. S., et al., *The SmtB/ArsR family of metalloregulatory transcriptional repressors: structural insights into prokaryotic metal resistance*. FEMS Microbiology Reviews, 2003. **27**: p.131-143.
8. Conrad, T., et al., *Maximizing transcription of nucleic acids with efficient T7 promoters*. Commun. Biol., 2020. **3**(1):p. 439.
