# Supplementary material for: Programming cell-free biosensors with DNA strand displacement circuits
Source: Nat Chem Biol. 2022 Feb 17;18(4):385–93. doi: 10.1038/s41589-021-00962-9 (PMC8964419; doi:10.1038/s41589-021-00962-9)
Supplement: Supplementary file 2 — Reporting Summary [file 41589_2021_962_MOESM2_ESM.pdf]

Corresponding author(s): Julius B. Lucks

Last updated by author(s): Nov 29, 2021

Reporting Summary

Nature Portfolio wishes to improve the reproducibility of the work that we publish. This form provides structure for consistency and transparency in reporting. For further information on Nature Portfolio policies, see our Editorial Policies and the Editorial Policy Checklist.

Statistics

For all statistical analyses, confirm that the following items are present in the figure legend, table legend, main text, or Methods section.

n/a	Confirmed	
☐	☒	The exact sample size (*n*) for each experimental group/condition, given as a discrete number and unit of measurement
☐	☒	A statement on whether measurements were taken from distinct samples or whether the same sample was measured repeatedly
☐	☒	The statistical test(s) used AND whether they are one- or two-sided<br>*Only common tests should be described solely by name; describe more complex techniques in the Methods section.*
☒	☐	A description of all covariates tested
☒	☐	A description of any assumptions or corrections, such as tests of normality and adjustment for multiple comparisons
☐	☒	A full description of the statistical parameters including central tendency (e.g. means) or other basic estimates (e.g. regression coefficient) AND variation (e.g. standard deviation) or associated estimates of uncertainty (e.g. confidence intervals)
☐	☒	For null hypothesis testing, the test statistic (e.g. F, t, r) with confidence intervals, effect sizes, degrees of freedom and P value noted<br>*Give P values as exact values whenever suitable.*
☒	☐	For Bayesian analysis, information on the choice of priors and Markov chain Monte Carlo settings
☒	☐	For hierarchical and complex designs, identification of the appropriate level for tests and full reporting of outcomes
☒	☐	Estimates of effect sizes (e.g. Cohen's *d*, Pearson's *r*), indicating how they were calculated

Our web collection on statistics for biologists contains articles on many of the points above.

Software and code

Policy information about availability of computer code

Data collection	BioTek Gen5 v2.04 (microplate reader software)<br>BioRad Image Lab Touch Software 1.2.0.12 (ChemiDoc imaging system software)<br>Spicy.Integrate Package in Python 3.7.6<br>Github link for the ODE codes used in this manuscript: https://git.io/Jtlh1
Data analysis	Microsoft Excel v16.45<br>Fiji - ImageJ v2.1.0/1.53c

For manuscripts utilizing custom algorithms or software that are central to the research but not yet described in published literature, software must be made available to editors and reviewers. We strongly encourage code deposition in a community repository (e.g. GitHub). See the Nature Portfolio guidelines for submitting code & software for further information.

Data

Policy information about availability of data

All manuscripts must include a data availability statement. This statement should provide the following information, where applicable:

- Accession codes, unique identifiers, or web links for publicly available datasets
- A description of any restrictions on data availability
- For clinical datasets or third party data, please ensure that the statement adheres to our policy

All data presented in this manuscript are available as Source Data and as Supplementary Data. All Source Data as well as Supplementary Data 2 and 3 are also deposited in Mendeley Data (doi: 10.17632/hr3j3yztxb.1). All plasmids used in this manuscript are available in Addgene with the identifiers 140371, 140374, 140391 and 140395.

Field-specific reporting

Please select the one below that is the best fit for your research. If you are not sure, read the appropriate sections before making your selection.

☒ Life sciences ☐ Behavioural & social sciences ☐ Ecological, evolutionary & environmental sciences

For a reference copy of the document with all sections, see nature.com/documents/nr-reporting-summary-flat.pdf

Life sciences study design

All studies must disclose on these points even when the disclosure is negative.

Sample size	No sample size calculation was used. Sample size of n=3 was chosen for all quantitative fluorescence measurements based on similar cell-free / in vitro studies. While polyacrylamide gel electrophoresis experiments were performed three times, one representative of each analysis was included in the manuscript based on similar in vitro studies that involve PAGE analysis. All individual data points are shown in the figures, and total sample size spanning all independent experiments is reported in the figure captions.
Data exclusions	No data on presented systems were excluded from the analyses. For the polyacrylamide gel images shown in Extended Data 1b, f, we did test another condition, but did not discuss it in the manuscript as it was irrelevant to the claims made from the gel analysis. The uncropped, unprocessed gel images in Source Data as well as Supplementary Data 2 include this condition because all conditions tested were run alongside, but not discussed in the manuscript.
Replication	All microplate reader experiments were performed three times by at least one co-author and are all presented in the manuscript. All polyacrylamide gel electrophoresis experiments were performed three times by at least one co-author, and one representatives of the replicates are presented in the manuscript. All attempts at replication were successful.
Randomization	While randomization is not relevant to this study since our biochemical reactions are handled uniformly, the same data analysis procedure was applied to all samples of the same type.
Blinding	Blinding is not relevant to this study since the results presented are based on objective description of our novel in vitro biosensing technology, and are therefore not subject to human biases.

Reporting for specific materials, systems and methods

We require information from authors about some types of materials, experimental systems and methods used in many studies. Here, indicate whether each material, system or method listed is relevant to your study. If you are not sure if a list item applies to your research, read the appropriate section before selecting a response.

Materials & experimental systems

n/a	Involved in the study	
☒	☐	Antibodies
☒	☐	Eukaryotic cell lines
☒	☐	Palaeontology and archaeology
☒	☐	Animals and other organisms
☒	☐	Human research participants
☒	☐	Clinical data
☒	☐	Dual use research of concern

Methods

n/a	Involved in the study	
☒	☐	ChIP-seq
☒	☐	Flow cytometry
☒	☐	MRI-based neuroimaging
