# Supplementary figures and images for: Programming cell-free biosensors with DNA strand displacement circuits
Source: Nat Chem Biol. 2022 Feb 17;18(4):385–93. doi: 10.1038/s41589-021-00962-9 (PMC8964419; doi:10.1038/s41589-021-00962-9)

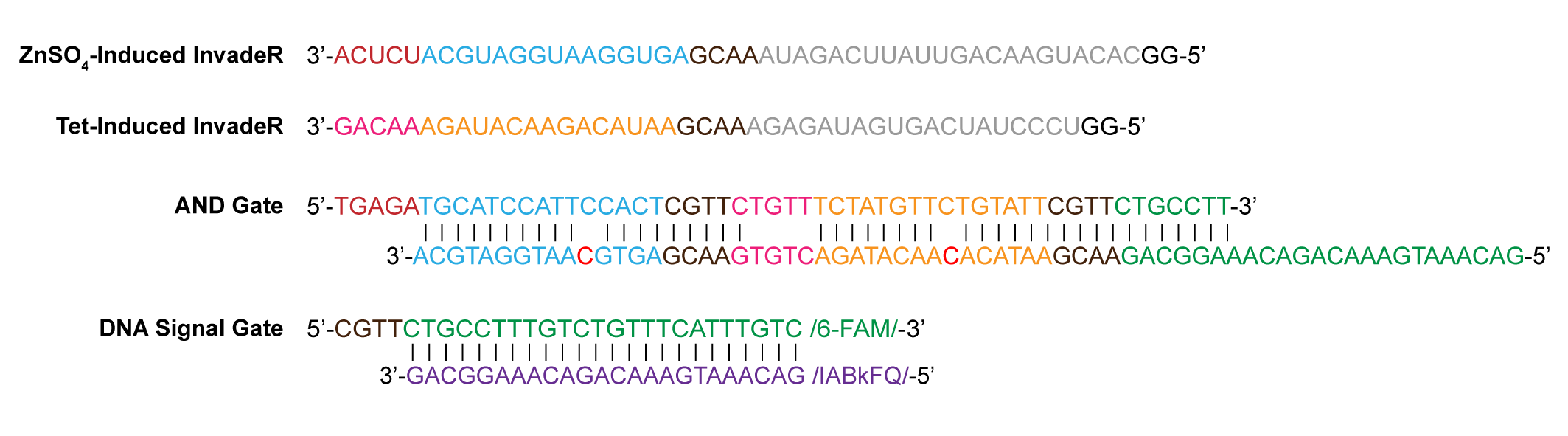

Supplement: Supplementary file 6 — Designs and sequences of all logic gates built in this study and their sources. [file 41589_2021_962_MOESM6_ESM.zip › Jung_TMSD_ROSALIND_Supp_Data_File4_Logic_Gate_Designs/AND/AND.tif]

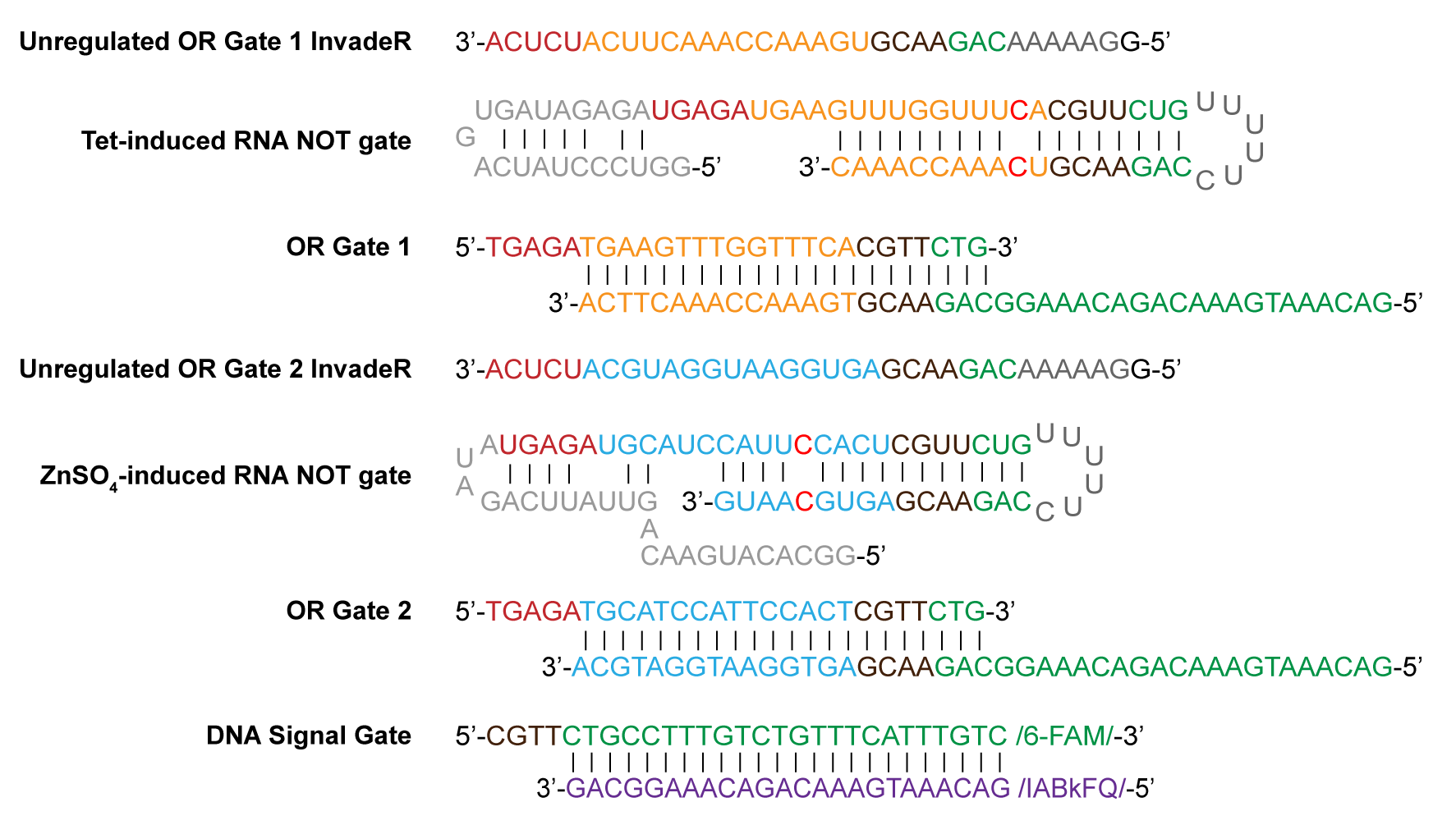

Supplement: Supplementary file 6 — Designs and sequences of all logic gates built in this study and their sources. [file 41589_2021_962_MOESM6_ESM.zip › Jung_TMSD_ROSALIND_Supp_Data_File4_Logic_Gate_Designs/NAND/NAND.tif]

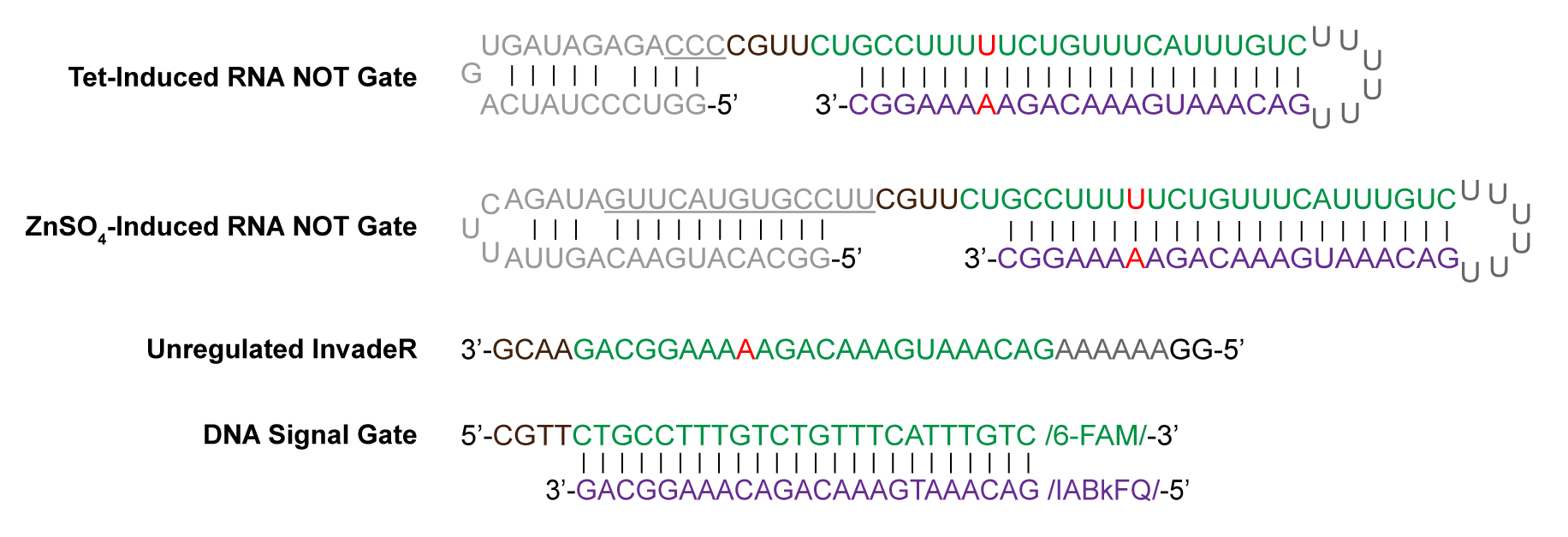

Supplement: Supplementary file 6 — Designs and sequences of all logic gates built in this study and their sources. [file 41589_2021_962_MOESM6_ESM.zip › Jung_TMSD_ROSALIND_Supp_Data_File4_Logic_Gate_Designs/NOR/NOR.tif]

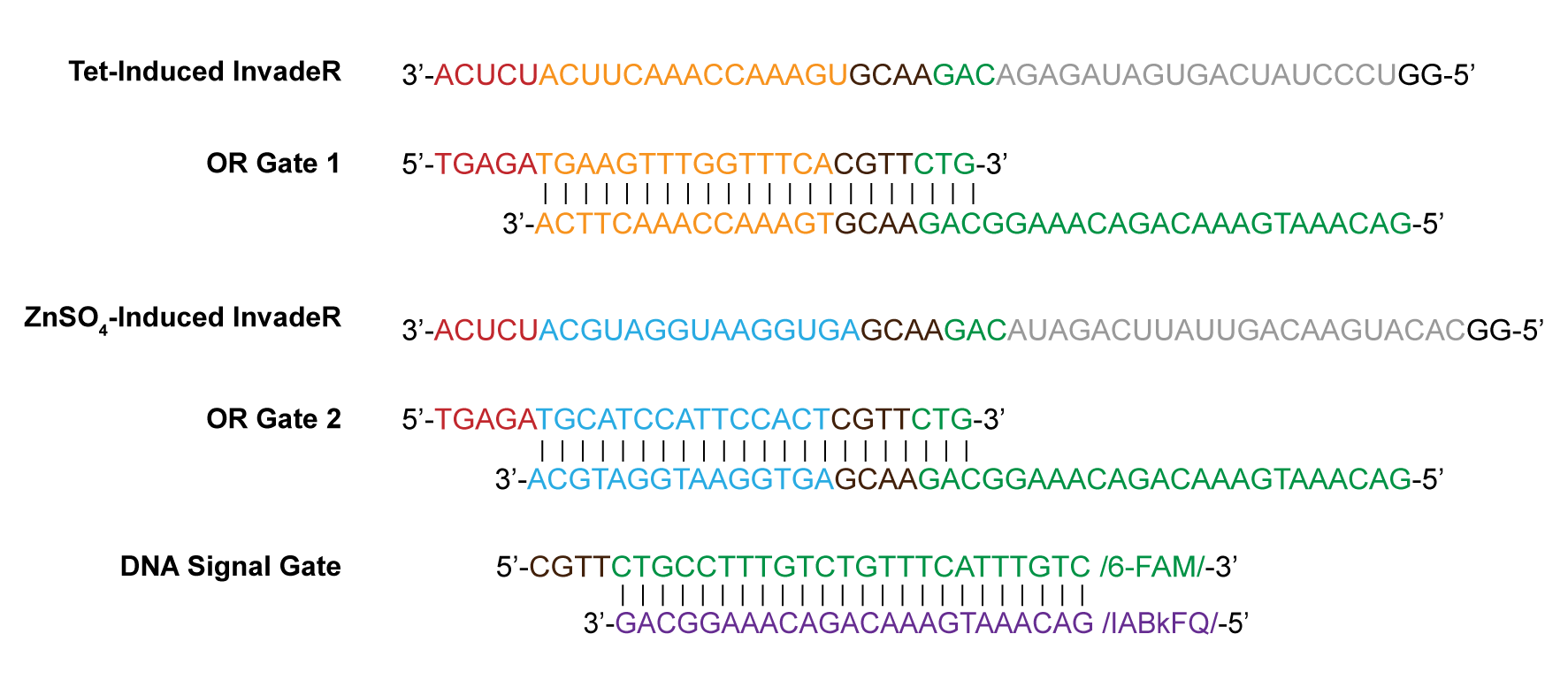

Supplement: Supplementary file 6 — Designs and sequences of all logic gates built in this study and their sources. [file 41589_2021_962_MOESM6_ESM.zip › Jung_TMSD_ROSALIND_Supp_Data_File4_Logic_Gate_Designs/OR/OR.tif]

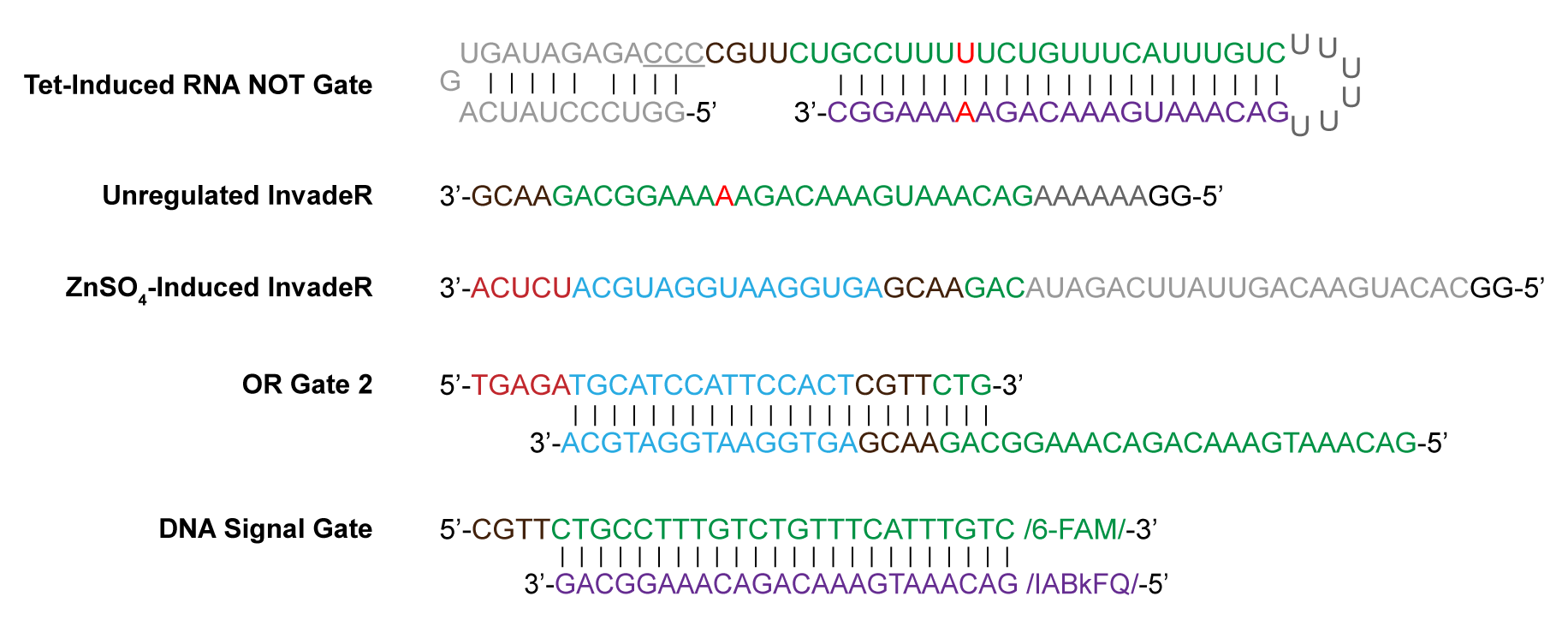

Supplement: Supplementary file 6 — Designs and sequences of all logic gates built in this study and their sources. [file 41589_2021_962_MOESM6_ESM.zip › Jung_TMSD_ROSALIND_Supp_Data_File4_Logic_Gate_Designs/IMPLY_v1/Tetracycline/IMPLY_v1-Tet.tif]

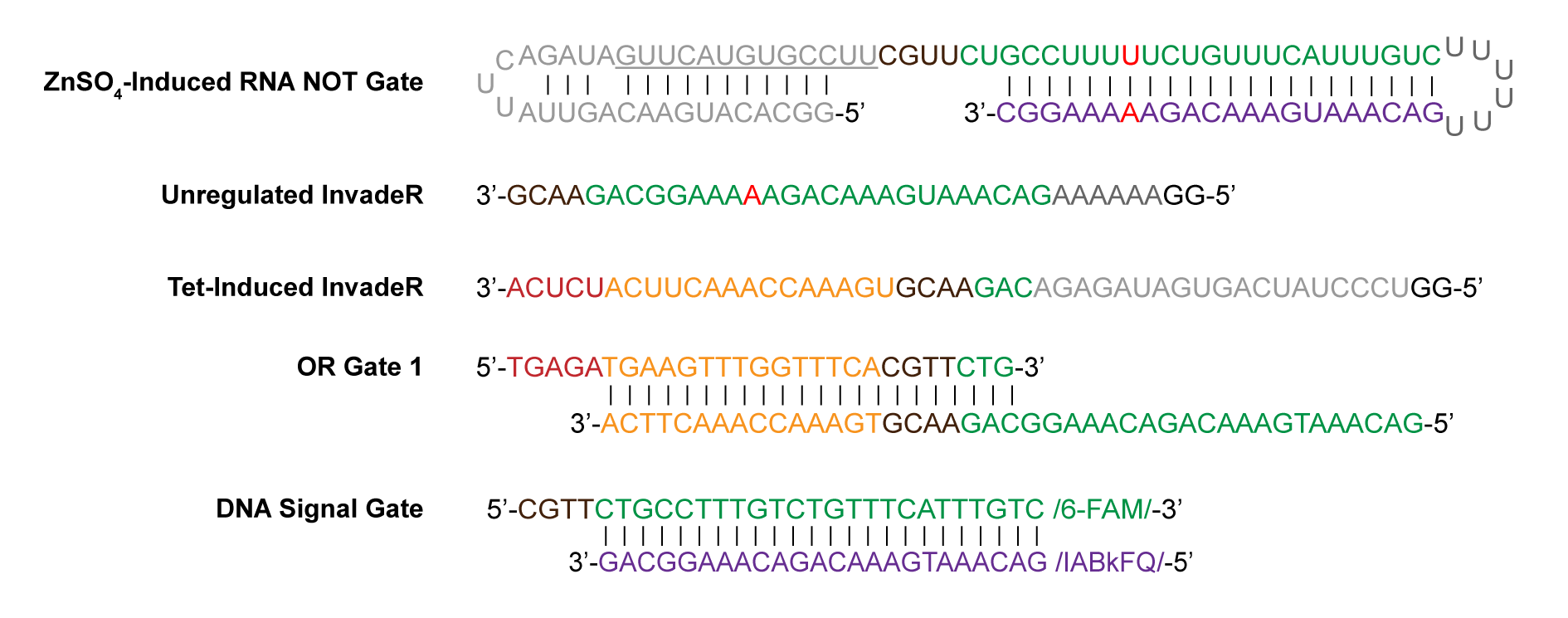

Supplement: Supplementary file 6 — Designs and sequences of all logic gates built in this study and their sources. [file 41589_2021_962_MOESM6_ESM.zip › Jung_TMSD_ROSALIND_Supp_Data_File4_Logic_Gate_Designs/IMPLY_v1/ZnSO4/IMPLY_v1-ZnSO4.tif]

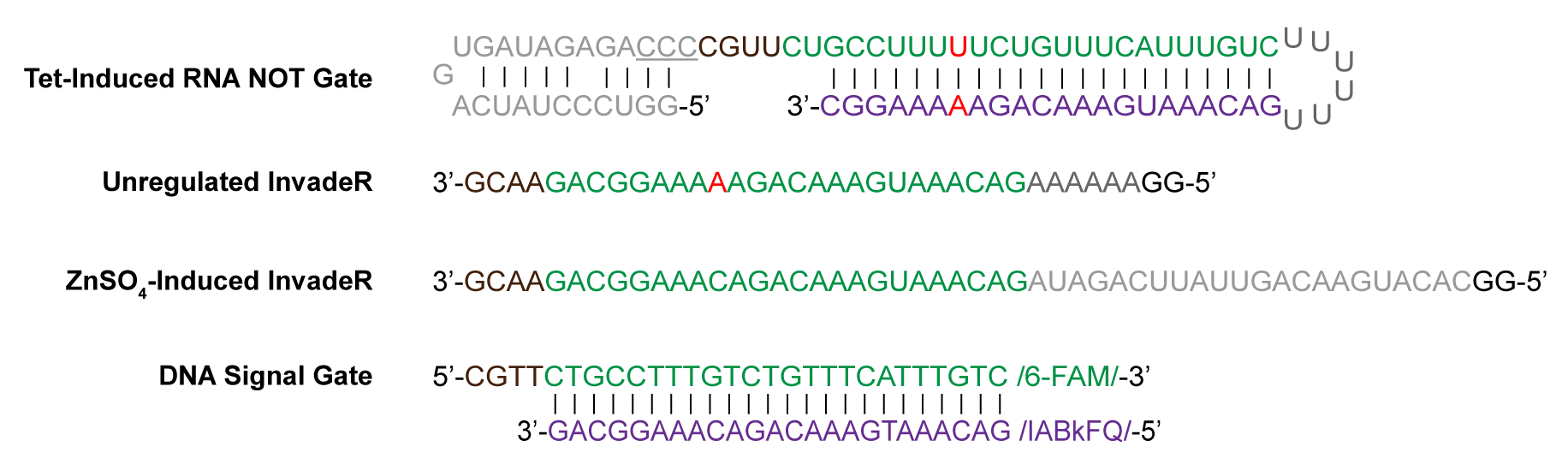

Supplement: Supplementary file 6 — Designs and sequences of all logic gates built in this study and their sources. [file 41589_2021_962_MOESM6_ESM.zip › Jung_TMSD_ROSALIND_Supp_Data_File4_Logic_Gate_Designs/IMPLY_v2/Tetracycline/IMPLY_v2-Tet.tif]

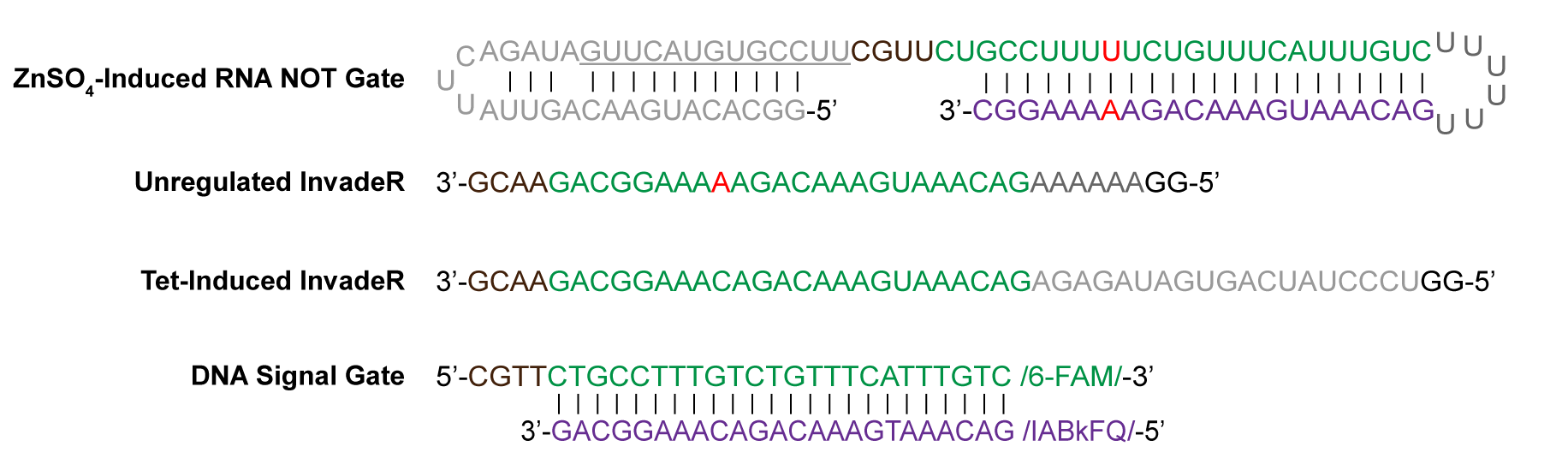

Supplement: Supplementary file 6 — Designs and sequences of all logic gates built in this study and their sources. [file 41589_2021_962_MOESM6_ESM.zip › Jung_TMSD_ROSALIND_Supp_Data_File4_Logic_Gate_Designs/IMPLY_v2/ZnSO4/IMPLY_v2-ZnSO4.tif]

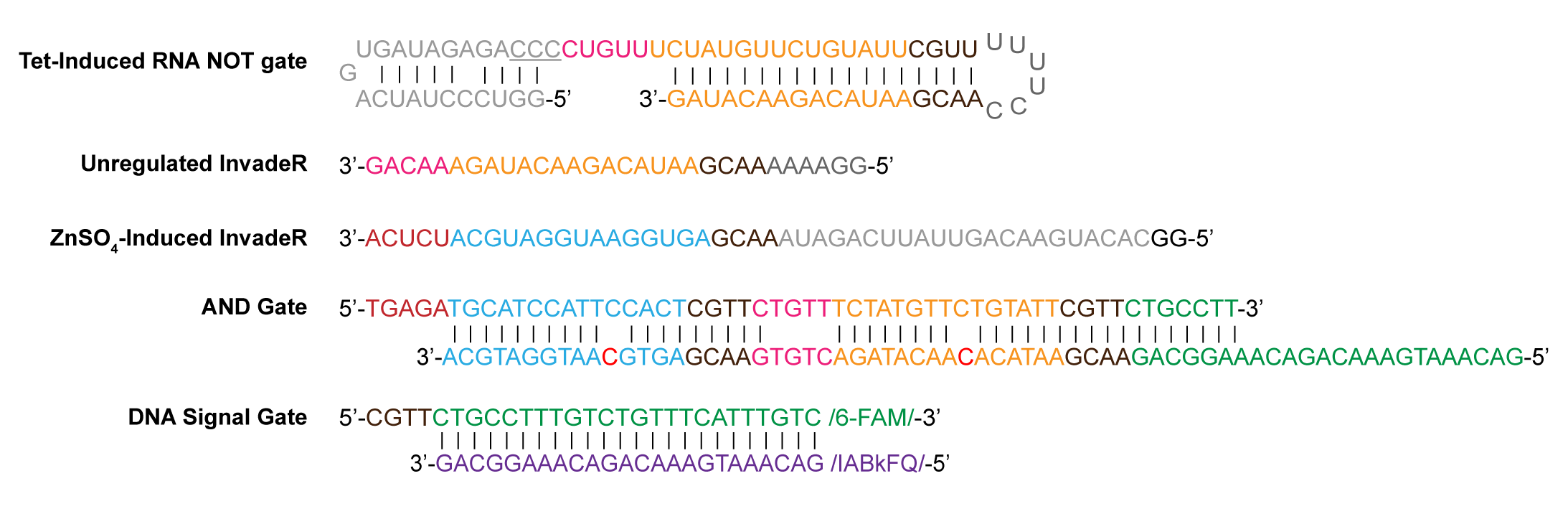

Supplement: Supplementary file 6 — Designs and sequences of all logic gates built in this study and their sources. [file 41589_2021_962_MOESM6_ESM.zip › Jung_TMSD_ROSALIND_Supp_Data_File4_Logic_Gate_Designs/NIMPLY/Tetracycline/NIMPLY-Tet.tif]

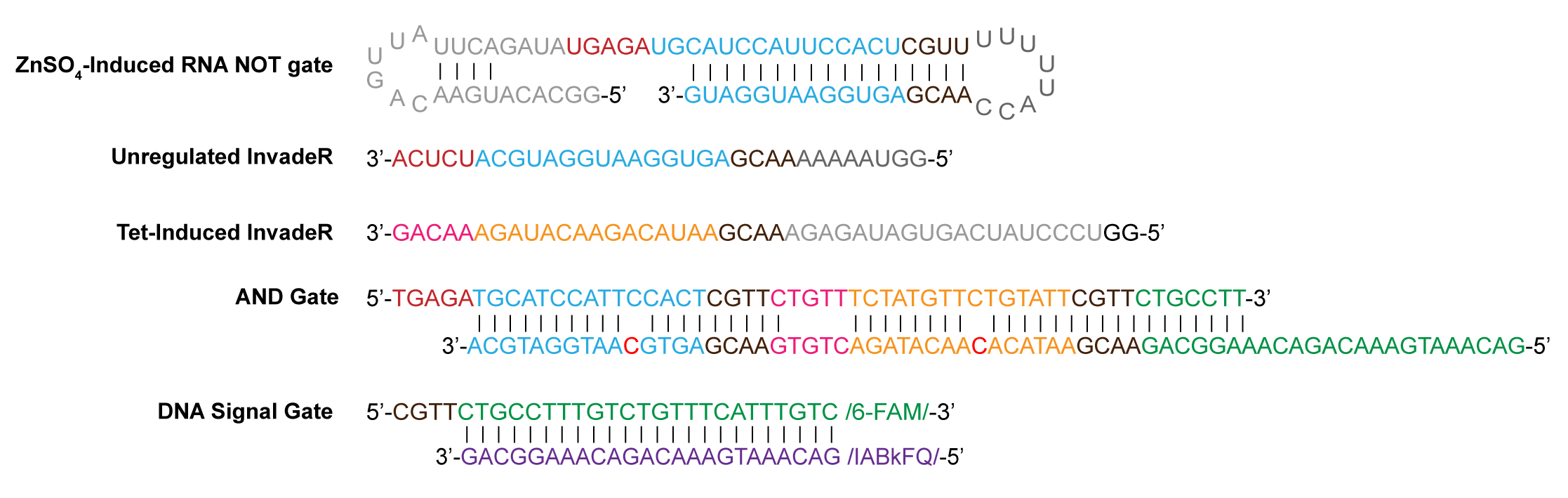

Supplement: Supplementary file 6 — Designs and sequences of all logic gates built in this study and their sources. [file 41589_2021_962_MOESM6_ESM.zip › Jung_TMSD_ROSALIND_Supp_Data_File4_Logic_Gate_Designs/NIMPLY/ZnSO4/NIMPLY-ZnSO4.tif]

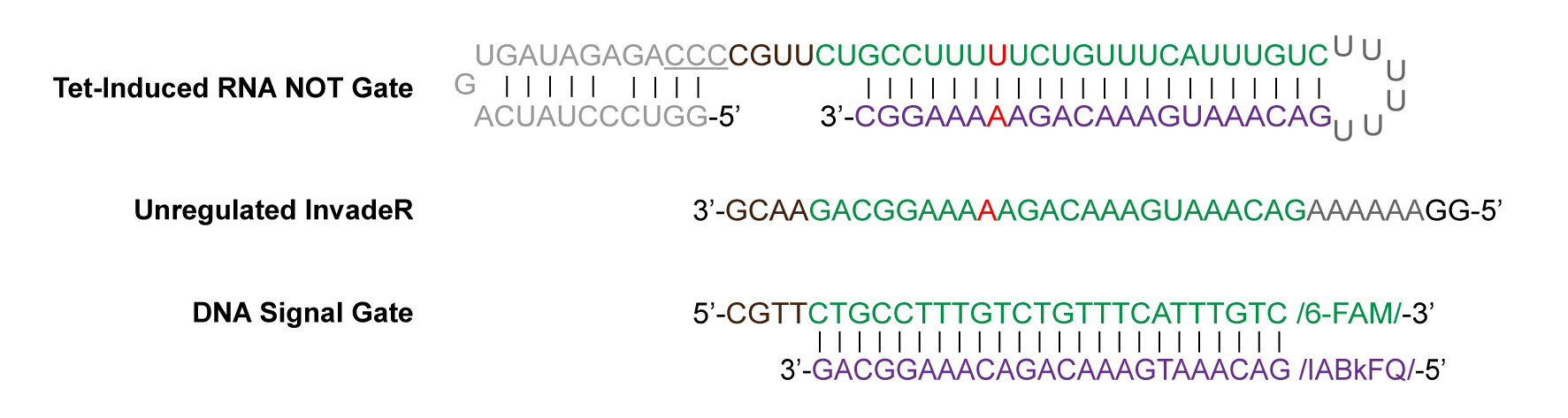

Supplement: Supplementary file 6 — Designs and sequences of all logic gates built in this study and their sources. [file 41589_2021_962_MOESM6_ESM.zip › Jung_TMSD_ROSALIND_Supp_Data_File4_Logic_Gate_Designs/NOT/Tetracycline/NOT-Tet.tif]

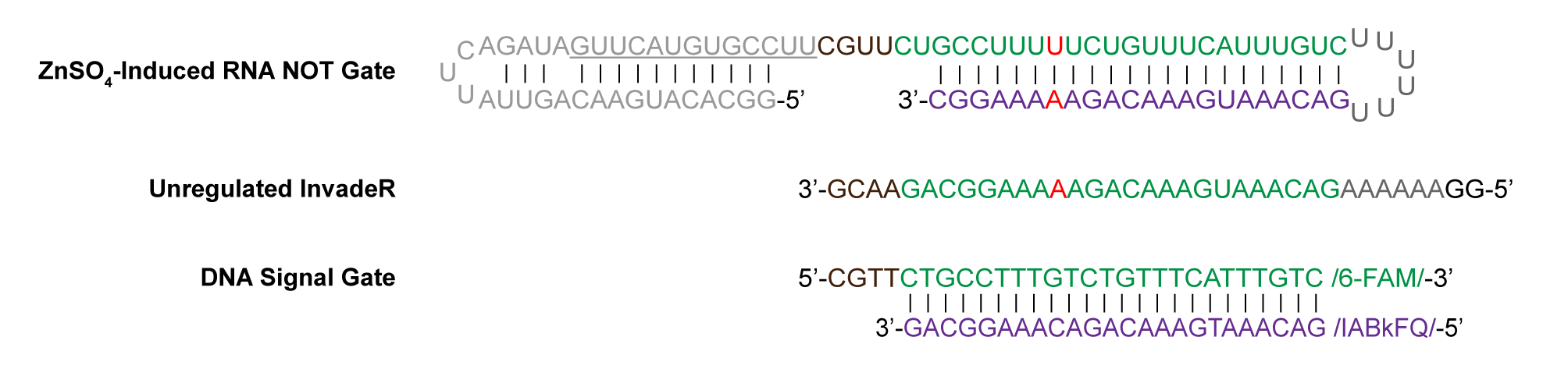

Supplement: Supplementary file 6 — Designs and sequences of all logic gates built in this study and their sources. [file 41589_2021_962_MOESM6_ESM.zip › Jung_TMSD_ROSALIND_Supp_Data_File4_Logic_Gate_Designs/NOT/ZnSO4/NOT-ZnSO4.tif]
