# Supplementary material for: Programming cell-free biosensors with DNA strand displacement circuits
Source: Nat Chem Biol. 2022 Feb 17;18(4):385–93. doi: 10.1038/s41589-021-00962-9 (PMC8964419; doi:10.1038/s41589-021-00962-9)
Supplement: Source Data Extended Data Fig. 1 — Unprocessed, uncropped urea–PAGE gels shown in Extended Data Fig. 1 and their short descriptions. [file 41589_2021_962_MOESM15_ESM.pdf]

"Programming Cell-Free Biosensors with DNA Strand Displacement Circuits."
Jung, et al. (2021)

Image below shows the uncropped, unprocessed urea-PAGE gel image of data shown in **Extended Data 1c** and **Extended Data 1f** unmodified lanes. Lanes 4, 5, 6, 10 and 11 are not shown in the manuscript.

From left to right:

1. Low Molecular ssDNA Ladder (10, 15, 20, 25, 30, 35, 40, 45, 50, 60, 70, 80, 90, 100 nt)
2. (-) 3' toehold DNA signal gate
3. (+) 3' toehold DNA signal gate

7. Low Molecular ssDNA Ladder (10, 15, 20, 25, 30, 35, 40, 45, 50, 60, 70, 80, 90, 100 nt)
8. (-) 5' toehold DNA signal gate
9. (+) 5' toehold DNA signal gate

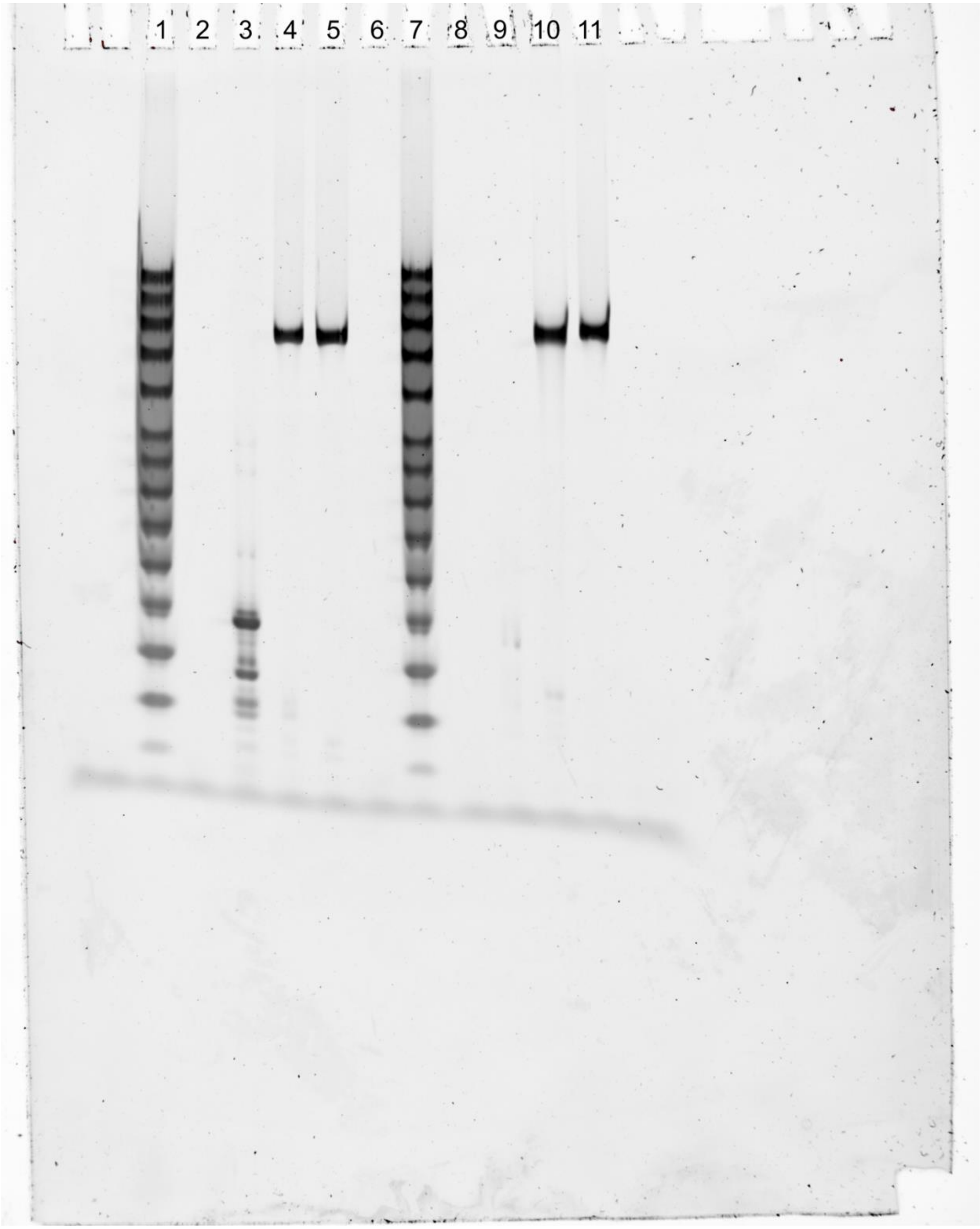

1 2 3 4 5 6 7 8 9 10 11

"Programming Cell-Free Biosensors with DNA Strand Displacement Circuits."
Jung, et al. (2021)

Image below shows the uncropped, unprocessed urea-PAGE gel image of 2'-O-methylation modification data shown in **Extended Data 1f**. Lanes 1–6, 10 and 11 are not shown in the manuscript. The 3' toehold DNA signal gate without 2'-O-methylation modification data shown are the same gel image used in **Extended Data 1c**.

From left to right:

7. Low Molecular ssDNA Ladder (10, 15, 20, 25, 30, 35, 40, 45, 50, 60, 70, 80, 90, 100 nt)
8. (-) 3' toehold DNA signal gate with 2'O-methylation modification
9. (+) 3' toehold DNA signal gate with 2'O-methylation modification

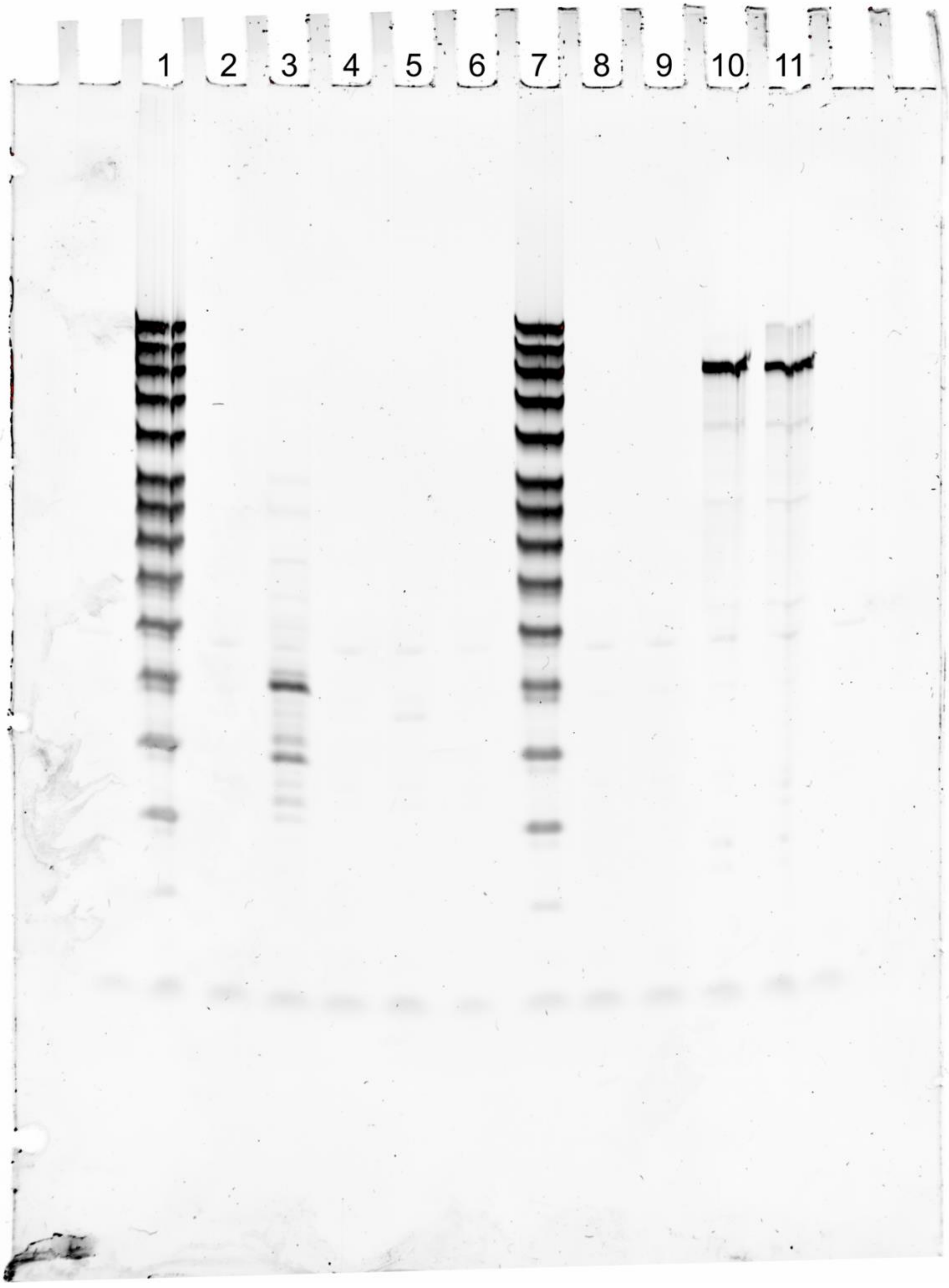
1 2 3 4 5 6 7 8 9 10 11
