# Supplementary material for: Programming cell-free biosensors with DNA strand displacement circuits
Source: Nat Chem Biol. 2022 Feb 17;18(4):385–93. doi: 10.1038/s41589-021-00962-9 (PMC8964419; doi:10.1038/s41589-021-00962-9)
Supplement: Source Data Extended Data Fig. 2 — Unprocessed, uncropped urea–PAGE gel shown in Extended Data Fig. 2 and its short description. [file 41589_2021_962_MOESM17_ESM.pdf]

"Programming Cell-Free Biosensors with DNA Strand Displacement Circuits."
Jung, et al. (2021)

Image below shows the uncropped, unprocessed urea-PAGE gel image of data shown in **Extended Data 2c**.

From left to right:
1. 0.25 ng of Standard RNA
2. 0.5 ng of Standard RNA
3. 1 ng of Standard RNA
4. 2 ng of Standard RNA
5. 4 ng of Standard RNA
6. 6 ng of Standard RNA
7. 8 ng of Standard RNA
8. 10 ng of Standard RNA
9. Low Molecular ssDNA Ladder (10, 15, 20, 25, 30, 40, 50, 60 nt)
10. InvadeR Variant 1 designed to strand-displace the 5' toehold DNA signal gate extracted from a 30-min long IVT reaction
11. InvadeR Variant 2 designed to strand-displace the 5' toehold DNA signal gate extracted from a 30-min long IVT reaction
12. InvadeR Variant 2 Strengthened designed to strand-displace the 5' toehold DNA signal gate extracted from a 30-min long IVT reaction
13. InvadeR Variant 3 designed to strand-displace the 5' toehold DNA signal gate extracted from a 30-min long IVT reaction
14. InvadeR Variant 3 Strengthened designed to strand-displace the 5' toehold DNA signal gate extracted from a 30-min long IVT reaction

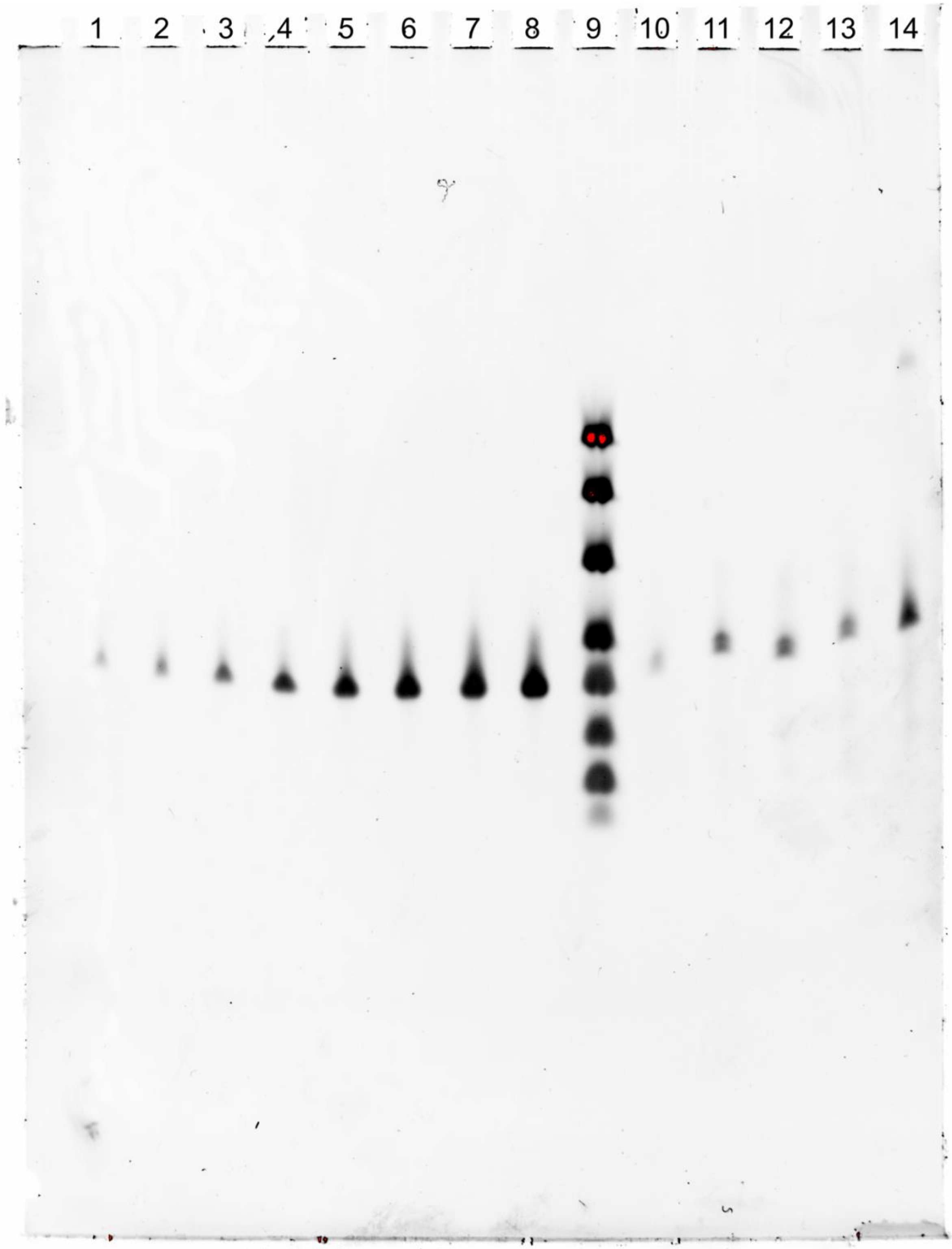
1
2
3
4
5
6
7
8
9
10
11
12
13
14
